# Structure of a bacterial RNA polymerase holoenzyme open promoter complex

**DOI:** 10.7554/eLife.08504

**Published:** 2015-09-08

**Authors:** Brian Bae, Andrey Feklistov, Agnieszka Lass-Napiorkowska, Robert Landick, Seth A Darst

**Affiliations:** 1 Laboratory for Molecular Biophysics, The Rockefeller University, New York, United States; 2 Edward A. Doisy Department of Biochemistry and Molecular Biology, Saint Louis University School of Medicine, St Louis, United States; 3 Department of Biochemistry, University of Wisconsin-madison, Madison, United States; 4 Department of Bacteriology, University of Wisconsin-Madison, Madison, United States; Harvard Medical School, United States

**Keywords:** open promoter compex, RNA polymerase, transcription, X-ray crystallography, *Thermus aquaticus*, *E. coli*, other

## Abstract

Initiation of transcription is a primary means for controlling gene expression. In bacteria, the RNA polymerase (RNAP) holoenzyme binds and unwinds promoter DNA, forming the transcription bubble of the open promoter complex (RPo). We have determined crystal structures, refined to 4.14 Å-resolution, of RPo containing *Thermus aquaticus* RNAP holoenzyme and promoter DNA that includes the full transcription bubble. The structures, combined with biochemical analyses, reveal key features supporting the formation and maintenance of the double-strand/single-strand DNA junction at the upstream edge of the −10 element where bubble formation initiates. The results also reveal RNAP interactions with duplex DNA just upstream of the −10 element and potential protein/DNA interactions that direct the DNA template strand into the RNAP active site. Addition of an RNA primer to yield a 4 base-pair post-translocated RNA:DNA hybrid mimics an initially transcribing complex at the point where steric clash initiates abortive initiation and σ^A^ dissociation.

**DOI:** http://dx.doi.org/10.7554/eLife.08504.001

## eLife digest

Inside cells, molecules of double-stranded DNA encode the instructions needed to make proteins. To make a protein, the two strands of DNA that make up a gene are separated and one strand acts as a template to make molecules of messenger ribonucleic acid (or mRNA for short). This process is called transcription. The mRNA is then used as a template to assemble the protein. An enzyme called RNA polymerase carries out transcription and is found in all cells ranging from bacteria to humans and other animals.

Bacteria have the simplest form of RNA polymerase and provide an excellent system to study how it controls transcription. It is made up of several proteins that work together to make RNA using DNA as a template. However, it requires the help of another protein called sigma factor to direct it to regions of DNA called promoters, which are just before the start of the gene. When RNA polymerase and the sigma factor interact the resulting group of proteins is known as the RNA polymerase ‘holoenzyme’.

Transcription takes place in several stages. To start with, the RNA polymerase holoenzyme locates and binds to promoter DNA. Next, it separates the two strands of DNA and exposes a portion of the template strand. At this point, the DNA and the holoenzyme are said to be in an ‘open promoter complex’ and the section of promoter DNA that is within it is known as a ‘transcription bubble’. However, it is not clear how RNA polymerase holoenzyme interacts with DNA in the open promoter complex.

Bae, Feklistov et al. have now used X-ray crystallography to reveal the three-dimensional structure of the open promoter complex with an entire transcription bubble from a bacterium called *Thermus aquaticus.* The experiments show that there are several important interactions between RNA polymerase holoenzyme and promoter DNA. In particular, the sigma factor inserts into a region of the DNA at the start of the transcription bubble. This rearranges the DNA in a manner that allows the DNA to be exposed and contact the main part of the RNA polymerase. If the holoenyzyme fails to contact the DNA in this way, the holoenzyme does not bind properly to the promoter and transcription does not start.

These findings build on previous work to provide a detailed structural framework for understanding how the RNA polymerase holoenzyme and DNA interact to form the open promoter complex. Another study by Bae et al.—which involved some of the same researchers as this study—reveals how another protein called CarD also binds to DNA at the start of the transcription bubble to stabilize the open promoter complex.

**DOI:** http://dx.doi.org/10.7554/eLife.08504.002

## Introduction

Transcription initiation is a major control point of gene expression. The initiation process is best understood in the bacterial system (Saecker et al., 2011) where the conserved ∼400 kD catalytic core of the RNA polymerase (RNAP or E, subunit composition α_2_ββ′ω) combines with the promoter-specificity factor σ^A^ to form the holoenzyme (Eσ^A^), which locates promoter DNA and unwinds 12–14 base pairs (bps) of the DNA duplex to yield the transcription-competent open promoter complex (RPo). In the presence of nucleotide substrates, RNA synthesis begins with the formation of an initial transcription complex (RP_ITC_). Before transitioning to a stable elongation complex, steric clash between the elongating RNA transcript and elements of σ set up abortive initiation, where the RNAP repeatedly generates and releases short transcripts without dissociating from the promoter (McClure et al., 1978; Murakami et al., 2002a; Goldman et al., 2009). Eventually, the transcript reaches a length of around 17 nt, where σ dissociation and the transition to the stable elongation complex begins (Nickels et al., 2005).

The architecture of Eσ^A^ recognition of the key −35 and −10 promoter elements was delineated by the structure of *Thermus aquaticus* (*Taq*) Eσ^A^ bound to an upstream fork (us-fork) promoter fragment, but the low resolution (6.5 Å) prevented the visualization of molecular details (Murakami et al., 2002b). Although high resolution crystal structures defined key, sequence-specific interactions of σ with the −35 element (Campbell et al., 2002), the melted −10 element (Feklistov and Darst, 2011), as well as with downstream promoter DNA in the context of holoenzyme (Zhang et al., 2012), these structures did not contain the full transcription bubble with the upstream double-strand/single-strand (ds/ss) DNA junction at the upstream edge of the −10 element where transcription bubble formation initiates.

**Figure 1.**
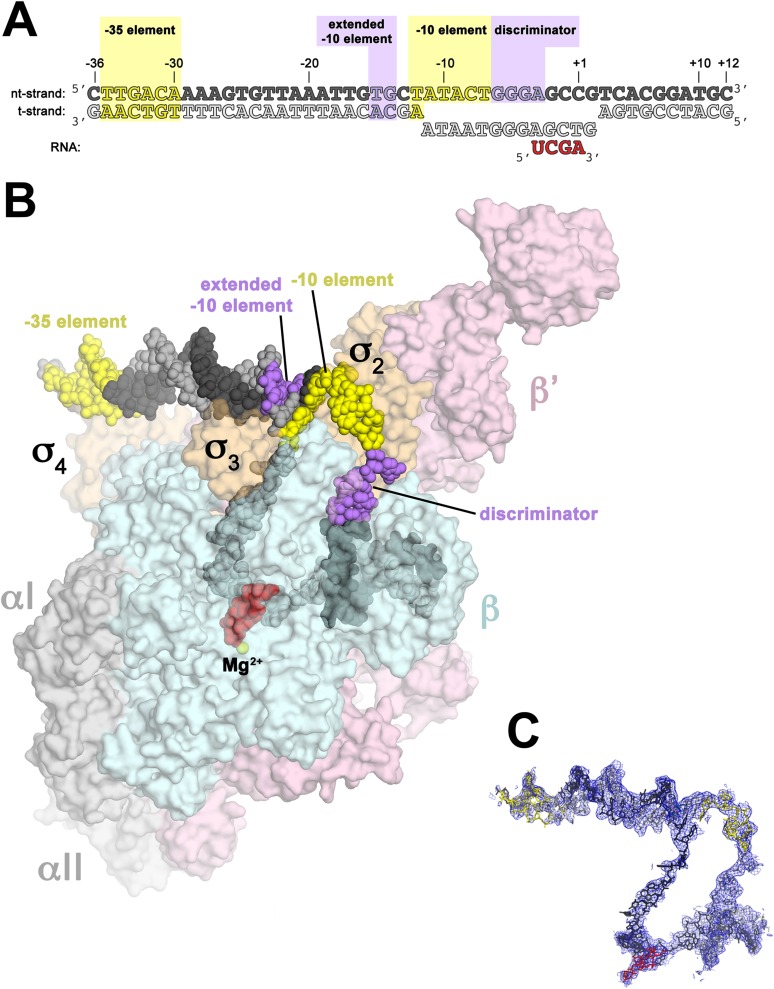
Structure of RPo. (**A**) Oligonucleotides used for RPo crystallization. The numbers above denote the DNA position with respect to the transcription start site (+1). The DNA sequence is derived from the full con promoter (Gaal et al., 2001). The −35 and −10 (Pribnow box) elements are shaded yellow, the extended −10 (Keilty and Rosenberg, 1987) and discriminator (Feklistov et al., 2006; Haugen et al., 2006) elements purple. The nt-strand DNA (top strand) is colored dark grey; t-strand DNA (bottom strand), light grey; RNA transcript, red. (**B**) Overall structure of RPo. The nucleic acids are shown as CPK spheres and color-coded as above. The *Taq* EΔ1.1σ^A^ is shown as a molecular surface (αI, αII, ω, grey; β, light cyan; β′, light pink; Δ1.1σ^A^, light orange), transparent to reveal the RNAP active site Mg^2+^ (yellow sphere) and the nucleic acids held inside the RNAP active site channel. (**C**) Electron density and model for RPo nucleic acids. Blue mesh, 2*F*_o_ − *F*_c_ maps for nucleic acids (contoured at 0.7σ). **DOI:** http://dx.doi.org/10.7554/eLife.08504.003

Structures of *Escherichia coli* (*Eco*) transcription initiation complexes containing a complete transcription bubble delineated the overall architecture of the full bubble, but the low resolution of the analyses (between 5.5 and 6 Å resolution) prevented a detailed description of protein/DNA interactions (Zuo and Steitz, 2015). Here, we determine crystal structures of *Taq* Eσ^A^ bound to an us-fork promoter fragment, as well as a complete RPo (Figure 1, Figure 1—figure supplement 1), refined using diffraction data extending to 4.00 and 4.14 Å-resolution, respectively (Table 1, Figure 1—figure supplement 2), allowing visualization of key features that stabilize the upstream edge of the transcription bubble. The results also reveal functionally relevant holoenzyme interactions with duplex DNA just upstream of the −10 element and potential protein/DNA interactions that direct the DNA template strand (t-strand) into the RNAP active site. Addition of an RNA primer to yield a 4-bp post-translocated RNA:DNA hybrid mimics RP_ITC_ at the point where steric clash initiates abortive initiation and σ^A^ dissociation (Murakami et al., 2002a; Kulbachinskiy and Mustaev, 2006).

**Figure 1—figure supplement 1.**
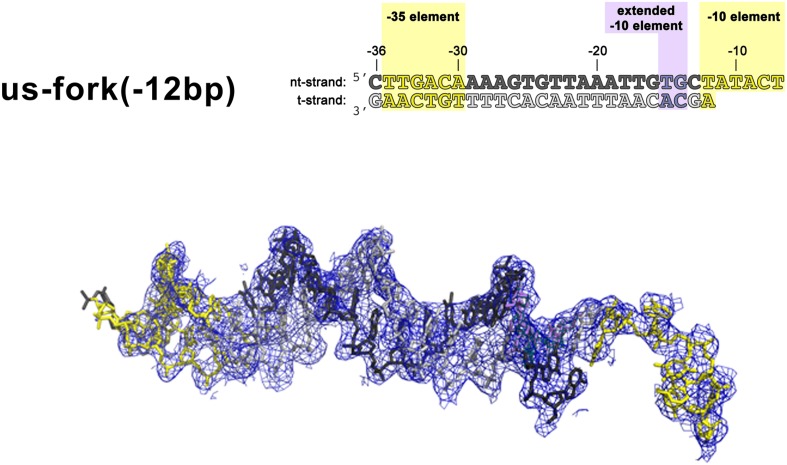
*(Left)* Synthetic oligonucleotides used for us-fork (−12 bp) crystallization. The numbers above the sequence denote the DNA position with respect to the transcription start site (+1). The DNA sequence is derived from the full con promoter (Gaal et al., 2001). The −35 and −10 (Pribnow box) elements are shaded yellow, the extended −10 (Keilty and Rosenberg, 1987) and discriminator (Feklistov et al., 2006; Haugen et al., 2006) elements purple. The nt-strand DNA (top strand) is colored dark grey; the t-strand DNA (bottom strand), light grey. (*Right*) Electron density and model for nucleic acids. Blue mesh, 2*F*_o_ − *F*_c_ maps for nucleic acids (contoured at 0.7σ). **DOI:** http://dx.doi.org/10.7554/eLife.08504.004

**Figure 1—figure supplement 2.**
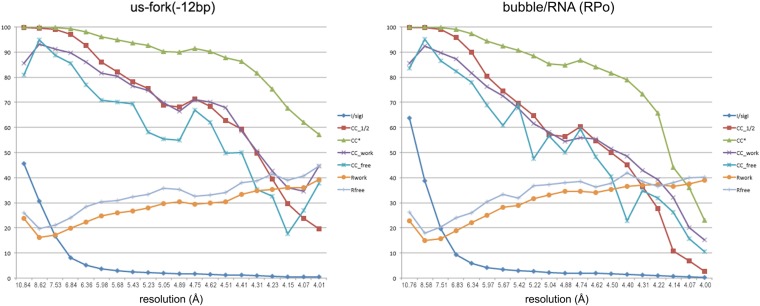
Data and model quality for us-fork (−12 bp) and RPo complexes. Plots relating data quality with model quality at 4.0 Å-resolution) using the Pearson correlation coefficient (CC) analysis described by Karplus and Diederichs (2012). CC1/2 (red squares) was determined from the unmerged diffraction data randomly divided in half. Since CC1/2 underestimates the information content of the data (since it's calculated by dividing the dataset in half), CC* was calculated from an analytical relation to estimate the information content of the full data (Karplus and Diederichs, 2012). CC* provides a statistic that assesses data quality as well and also allows direct comparison of crystallographic model quality and data quality on the same scale through CC_work_ and CC_free_, the standard and cross-validated correlations of the experimental intensities with the intensities calculated from the refined model. A CC_work_/CC_free_ smaller than CC* indicates that the model does not account for all of the signal in the data, meaning it is not overfit. Plotted also are the standard <*I*>/σ*I* for the diffraction data, as well as the *R*_work_/*R*_free_ for the refined models. (*Left*) Data for *Taq* EΔ1.1σ^A^/us-fork (−12 bp) at 4.0 Å-resolution. (*Right*) Data for *Taq* EΔ1.1σ^A^ RPo (with 4-nt RNA primer) at 4.0 Å-resolution. **DOI:** http://dx.doi.org/10.7554/eLife.08504.005

**Figure 1—figure supplement 3.**
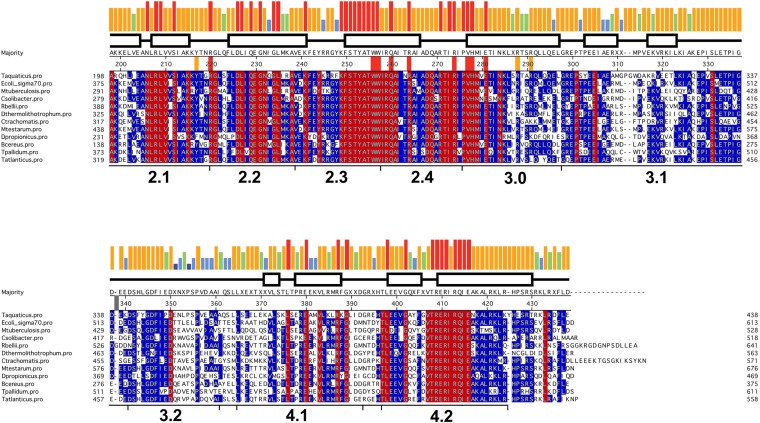
Sequence alignment of regions 2–4 of selected bacterial RNAP primary (Group I) σ subunits. Sequence alignment of regions 2–4 (Lonetto et al., 1992) of bacterial RNAP primary (Group I) σ subunits. The sequences shown were selected from diverse phyla/groups taken from a much larger alignment of 1002 sequences. The σ^A^ sequences shown are from the following organisms chosen to represent the preceding phylum/group: Deinococcus-Thermus, *Thermus aquaticus*; γ-Proteobacteria, *Escherichia coli*; Actinobacteria, *Mycobacterium tuberculosis*; Acidobacteria-*Candidatus Solibacter usitatus*; α-Proteobacteria, *Rickettsia belli*; Aquificae, *Desulfurobacterium thermolithotrophum*; Chlamydae, *Chlamydae trachomatis*; Cyanobacteria, *Mastigocoleus testarum*; δ*-*Proteobacteria; *Desulfobulbus propionicus*; Firmicutes, *Bacillus cereus*; Spirochaetes, *Treponema pallidum*; Thermodesulfobacteria, *Thermodesulfatator atlanticus*. The sequences are shaded according to conservation within the sub-alignment; red shading indicates 100% identity, blue shading indicates >50% identity. The histogram at the top represents the sequence conservation within the entire 1002 sequence alignment (red bar, 100% identity; orange, 83–99%; green, 68–83%; blue, 50–67%). **DOI:** http://dx.doi.org/10.7554/eLife.08504.006

**Table 1.** Table of crystallographic statistics **DOI:** http://dx.doi.org/10.7554/eLife.08504.007

*Taq* EΔ1.1σ^A^ +	Us-fork (−12 bp)	Us-fork (−11 bp)	Bubble/RNA (RPo)	Bubble
Data collection				
Space group	*P*4_3_2_1_2	*P*4_3_2_1_2	*P*4_3_2_1_2	*P*4_3_2_1_2
Combined datasets	3	4	10	4
Cell dimensions				
*a* (Å)	289.87	288.23	289.26	290.76
*b* (Å)	289.87	288.23	289.26	290.76
*c* (Å)	537.36	535.25	536.60	540.84
Wavelength (Å)	1.075	1.075	1.075	1.075
Resolution (Å)	50.03–4.01 (4.143–4.01)†	49.43–4.60 (4.76–4.60)†	34.96–4.14 (4.29–4.14)†	40.00–4.74 (4.91–4.74)†
Total reflections	2,192,774 (167,274)	1,268,008 (123,590)	5,022,989 (367,167)	1,849,900 (143,237)
Unique reflections	185,025 (18,323)	125,012 (11,043)	172,210 (16,966)	116,874 (8115)
Multiplicity	11.5 (9.1)	10.1 (10.1)	29.2 (21.6)	15.8 (12.7)
Completeness (%)	99.9 (98.6)	99.0 (100.00)	100 (99.8)	99.6 (97.0)
<*I*>/σ*I*	6.68 (0.43)	5.57 (0.60)	9.4 (0.8)	8.11 (0.81)
Wilson B-factor (Å^2^)	133.90	154.68	101.16	196.78
*R*_pim_‡	0.173 (2.136)	0.238 (1.816)	0.207 (1.264)	0.177 (2.047)
CC1/2§	0.988 (0.219)	0.975 (0.323)	0.983 (0.157)	0.974 (0.205)
CC*§	0.997 (0.601)	0.994 (0.698)	0.996 (0.521)	0.993 (0.584)
Anisotropic scaling B-factors¶				
a*, b* (Å^2^)	18.19	22.15	15.44	20.96
c* (Å^2^)	−36.37	−44.3	−30.88	−41.92
Refinement				
*R*_work_/*R*_free_	0.2531/0.2961 (0.3712/0.4188)	0.2446/0.2800 (0.3464/0.3726)	0.270/0.308 (0.358/0.371)	–
CC_work_/CC_free_§	0.918/0.900 (0.373/0.300)	0.923/0.904 (0.438/0.293)	0.897/0.890 (0.343/0.280)	–
No. atoms	56,478	56,501	58,279	–
Macromolecule	56,472	56,495	58,273	–
Ligand/ion	6	6	6	–
Water	0	0	0	–
Protein residues	6871	6871	6875	–
*B*-Factors				
Protein	139.60	175.65	137.7	–
Ligand/ion	169.70	175.69	134.4	–
R.m.s deviations				
Bond lengths (Å)	0.004	0.005	0.003	–
Bond angles (°)	0.91	1.12	0.80	–
Clashscore	11.91	22.89	12.88	–
Ramachandran favored (%)	94	88	92	–
Ramachandran outliers (%)	0.41	0.83	0.23	–

† Values in parentheses are for highest-resolution shell.

‡ (Diederichs and Karplus, 1997).

§ (Karplus and Diederichs, 2012).

¶ As determined by the UCLA MBI Diffraction Anisotropy Server (http://services.mbi.ucla.edu/anisoscale/).

## Results

### Overall structure of *Taq* RPo

We combined *Taq* EΔ1.1σ^A^ (Δ1.1σ^A^: *Taq* σ^A^ lacking the N-terminal region 1.1, which is dispensable for in vitro transcription. Region 1.1 is not expected to alter protein/DNA interactions in RPo) with us-fork promoter DNA, which contains a ds −35 element and a mostly ss −10 element (Figure 1—figure supplement 1). The resulting complex (423 kD) was crystallized and diffraction data were collected and analyzed (Table 1). The structure was determined by molecular replacement, which identified two complexes per asymmetric unit, and refined using data extending to 4 Å-resolution (Table 1, Figure 1—figure supplement 2). The solvent content of the crystals was 82% and examination of the crystal packing revealed space for the expected position of additional promoter DNA. We therefore formed a complete RPo by combining *Taq* EΔ1.1σ^A^ with a duplex promoter DNA scaffold (−36 to +12 with respect to the transcription start site at +1) but with a non-complementary transcription bubble generated by altering the sequence of the t-strand DNA from −11 to +2. RPo crystallized in the same habit and diffraction data were analyzed to 4.7 Å-resolution (Table 1). In the resulting electron density maps, most of the ss t-strand DNA was poorly ordered and unable to be modeled. To stabilize the t-strand DNA, we added an RNA primer complementary to the ss t-strand DNA from +1 to −3, yielding a 4 bp RNA:DNA hybrid (Figure 1A). We crystallized the resulting complex (437 kD, which we call RPo hereafter), collected and analyzed diffraction data, and refined the structure using reflections to a minimum Bragg spacing of 4.14 Å (Table 1, Figure 1—figure supplement 2). In RPo, good electron density for all of the nucleic acids included in the scaffold was observed (Figure 1C). The protein/DNA contacts seen in the us-fork complex are essentially identical to the relevant subset of contacts in RPo.

The extensive protein/DNA interface in RPo buries 6300 Å^2^ of molecular surface (Figure 1B). Overall close contacts with the nucleic acids occur from −36 to −30 and −17 to +9, consistent with hydroxyl-radical footprinting of RPo on promoters (Schickor et al., 1990; Ross and Gourse, 2009). Protein/DNA interactions are absent in the −35/−10 spacer DNA from −29 to −18.

Despite the relatively low resolution of our analysis (Table 1), important protein side chain/nucleic acid interactions were resolved in electron density maps. Protein side chain/nucleic acid interactions specifically discussed in this paper are supported by unbiased simulated annealing omit maps shown for each case (see below). The protein side chain/nucleic acid interactions specifically discussed in this paper occur via conserved (often universally) residues of the RNAP β′ or σ^A^ subunits. The level of conservation of relevant β′ residues, determined from an alignment of 834 bacterial RNAP β′ subunit sequences (Lane and Darst, 2010) is tabulated in Table 2. An alignment of 1002 diverse σ^A^ sequences was constructed (Supplementary file 1; a sub-alignment of selected diverse sequences is shown in Figure 1—figure supplement 3) and the level of conservation of relevant σ^A^ residues is tabulated in Table 3.

**Table 2.** Conservation of RNAP β′ subunit residues **DOI:** http://dx.doi.org/10.7554/eLife.08504.008

Residue	% Identity*	Blosum62 score*, †	Distribution of residues from alignment*
β′Y34	99.5	0.976	Y 803; H 1; Q 1; F 2
β′R35	99.4	0.980	R 829; K 5

* Determined from an alignment of 834 bacterial RNAP β′ subunit sequences (Lane and Darst, 2010).

† Blosum62 score calculated by PFAAT (Johnson et al., 2003).

**Table 3.** Conservation of σ^A^ residues **DOI:** http://dx.doi.org/10.7554/eLife.08504.009

Residue	% Identity*	Blosum62 score*, †	Distribution of residues from alignment*
σ^A^ Y217	99.4	0.988	996 Y; 5 H; 1 F
σ^A^ R220	100	0.998	–
σ^A^ W256	100	0.998	–
σ^A^ W257	100	0.998	–
σ^A^ Q260	100	0.998	–
σ^A^ R264	100	0.998	–
σ^A^ R274	100	0.998	–
σ^A^ V277	100	0.998	–
σ^A^ H278	100	0.998	–
σ^A^ E281	100	0.998	–
σ^A^ R288	99.7	0.993	999 R; 3 K
σ^A^ R291	99.7	0.988	997 R; 2 K; 1 H; 1 S; 1 L

* Determined from an alignment of 1002 bacterial RNAP primary σ subunit sequences (Supplementary file 1).

† Blosum62 score calculated by PFAAT (Johnson et al., 2003).

### RNAP interacts with ds DNA just upstream of the −10 element and specifically recognizes the extended −10 element

Starting from the upstream end of the promoter DNA, the −35 element interacts exclusively with $\sigma_{4}^{\text{A}}$ in a manner consistent with the high-resolution (2.4 Å) structure of the isolated $\sigma_{4}^{\text{A}}/-35$ element complex (Campbell et al., 2002). The duplex DNA just upstream of the −10 element (−17 to −13) interacts with β′, $\sigma_{3}^{\text{A}}$, and $\sigma_{2}^{\text{A}}$ (Figure 1B).

**Figure 2.**
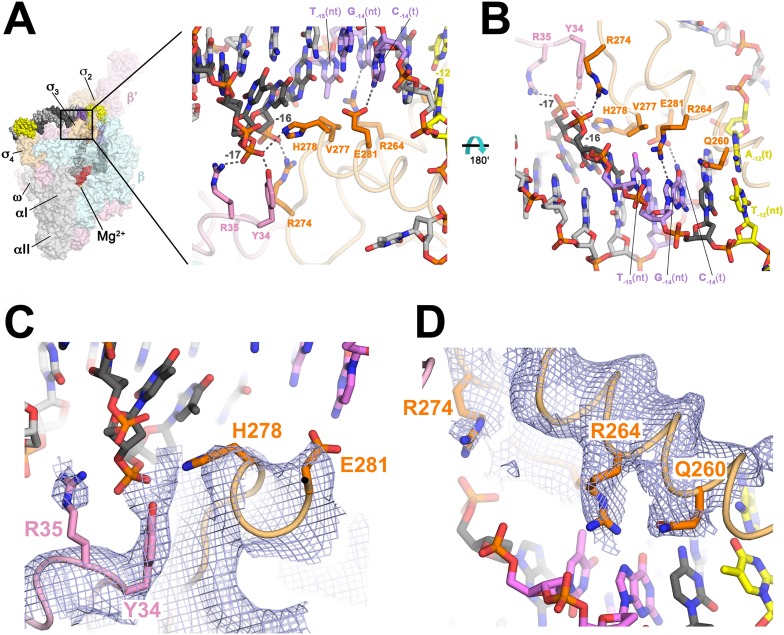
Protein interactions with duplex DNA upstream of the transcription bubble and recognition of the extended −10 element. (**A**) (*Left*) Overall view of RPo structure (similar to Figure 1B). The boxed area is magnified on the right. (*Right*) Magnified view showing protein interactions (β′ and σ^A^) with duplex DNA from −18 to −12. Proteins are shown as backbone worms (β′, light pink; σ^A^, light orange) with interacting side chains shown in stick format (β′, pink; σ^A^, orange). Likely polar interactions are denoted with grey dashed lines. (**B**) Same as (**A**) (*right*) but rotated 180° about the x-axis. (**C**) Similar view as (**A**) (*right*). Superimposed is the simulated annealing omit map (grey mesh, 2*F*_o_ − *F*_c_, contoured at 1σ), calculated from a model where the following protein segments were removed (β′ 33–36; σ^A^ 259–292) and shown only within 2 Å of omitted atoms. (**D**) Similar view as (**B**). Superimposed is the simulated annealing omit map (grey mesh, 2*F*_o_ − *F*_c_, contoured at 1σ), calculated from a model where the following protein segments were removed (β′ 33–36; σ^A^ 259–292) and shown only within 2 Å of omitted atoms. **DOI:** http://dx.doi.org/10.7554/eLife.08504.010

Previously, conserved residues of the β′-zipper (β′Y34 and, to a lesser extent, β′R35; Table 2) that contribute to RPo stability by interacting with duplex spacer DNA were identified (Yuzenkova et al., 2011). In the RPo structure, both β′Y34 and β′R35 are positioned to form polar interactions with the −17 nt-strand DNA (−17(nt)) phosphate (Figure 2A,C).

We observe many interactions of $\sigma_{3}^{\text{A}}$ and $\sigma_{2}^{\text{A}}$ with the duplex DNA just upstream of the transcription bubble (−17 to −12), predominantly with the nt-strand facing the holoenzyme (Figures 1B, 2). Conserved H278 and R274 of σ^A^ (corresponding to *Eco* σ^70^ H455 and R451; Figure 1—figure supplement 3; Table 3) are positioned to interact with the −17(nt) and −16(nt) phosphates, respectively (Figure 2). Substitution of either of these residues causes defects in promoter binding (Barne et al., 1997; Fenton et al., 2000; Singh et al., 2011).

Sequence-specific recognition of the duplex DNA upstream of the −10 element can occur through the extended −10 element (T_−15_G_−14_), which stabilizes RPo and can substitute for the −35 element (Keilty and Rosenberg, 1987). Conserved E281 of $\sigma_{3}^{\text{A}}$ (σ^70^ E458; Figure 1—figure supplement 3; Table 3) is positioned to recognize the −14 GC bp through a polar interaction with C_−14_(t), as predicted from allele-specific suppression genetics (Barne et al., 1997) (Figure 2A,C). G_−14_(nt) is also specifically recognized by conserved R264 (σ^70^ R441; Figure 1—figure supplement 3; Table 3) of $\sigma_{2}^{\text{A}}$ (Figure 2B,D). Substitutions in the corresponding amino acid position of an alternative σ cause defects in promoter recognition (Daniels et al., 1990). Methylation protection and interference indicates *Eco* Eσ^70^ makes close contacts with G_−14_(nt) on an extended −10 promoter (Minchin and Busby, 1993). Conserved V277 (σ^70^ V454; Figure 1—figure supplement 3; Table 3) may contact the T_−15_(nt) methyl group, possibly explaining the preference for T at this position (Figure 2B).

The primary role of σ_2_ in −10 element recognition was first uncovered when substitutions of invariant Q260 (σ^70^ Q437; Figure 1—figure supplement 3; Table 3) were shown to affect sequence-specific recognition of the −12 bp (Kenney et al., 1989; Waldburger et al., 1990). Modeling suggested that Q260 may H-bond with the major-groove edge of A_−12_(t) (Feklistov and Darst, 2011). However, in our structures, the amide group of the Q260 side chain points away from the major-groove edge of A_−12_(t) and cannot form H-bonds (Figure 2B,D). We suggest that Q260 may form base-specific H-bonds with the −12 bp in an intermediate during the pathway to RPo formation (Saecker et al., 2011), whereas our structures represent the final, transcription ready RPo, explaining the genetic data.

### Structural role of σ^A^ aromatic residues in forming and stabilizing the upstream ds/ss junction of the transcription bubble

**Figure 3.**
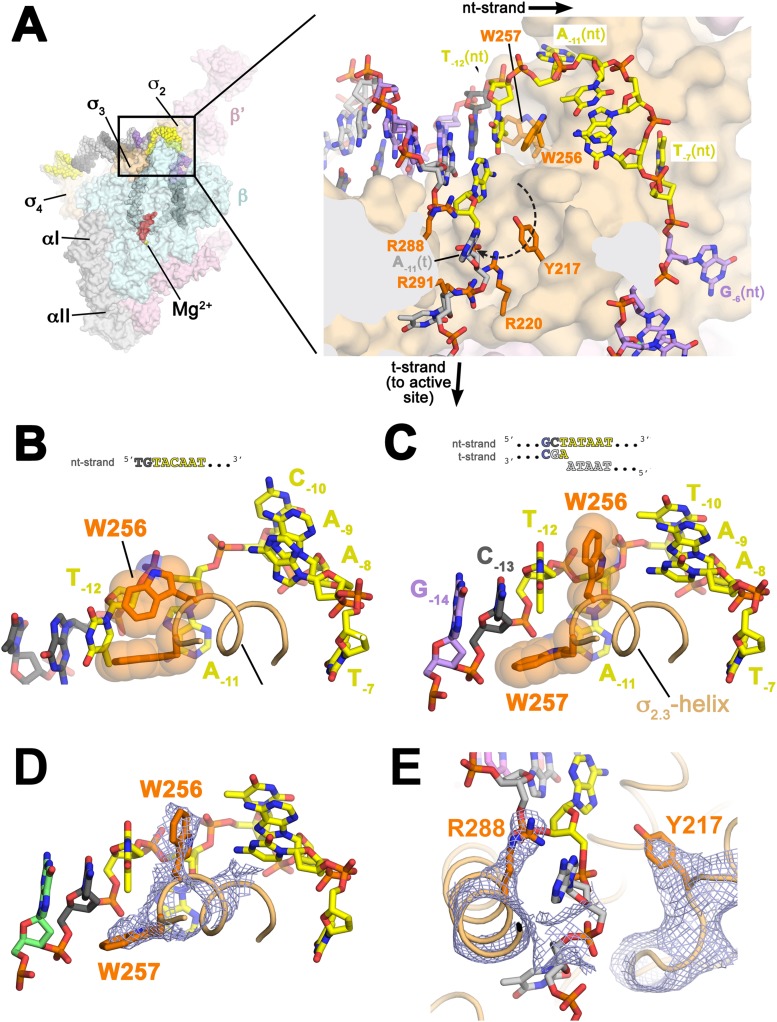
Structures maintaining the upstream ds/ss junction of the transcription bubble and directing the t-strand DNA to the RNAP active site. (**A**) (*Left*) Overall view of RPo structure (similar to Figure 1B). The boxed area is magnified on the right. (*Right*) Magnified view showing the upstream ds/ss junction of the transcription bubble in RPo (the RNAP β subunit, which obscures the view, has been removed). RNAP is shown as a molecular surface, except side chains of key σ^A^ residues (R217, R220, W256, R288, R291) are shown (orange). The orthogonal directions of the ss nt- and t-strand DNA following the upstream ds/ss junction are denoted by black arrows. The dashed, curved line denotes the potential path of the t-strand −11 base from its position in the duplex DNA (base-paired to A_−11_(nt)) to its position in the structure. (**B**) Structure of *Taq* $\sigma_{2}^{\text{A}}$ bound to the ss, nt-strand −10 element (PDB ID 3UGO) (Feklistov and Darst, 2011) showing the disposition of the universally conserved σ^A^ W-dyad (*Taq* σ^A^ W256/W257). Shown is the ss DNA from −14 to −7 (−10 element colored yellow), the $\sigma_{2.3}^{\text{A}}-\text{helix}$ (light orange) and the W-dyad (orange side chains with transparent CPK atoms). W256 makes an edge-on interaction with the face of W257, as observed in all other σ^70^/σ^A^ structures in many different contexts (Malhotra et al., 1996; Campbell et al., 2002; Vassylyev et al., 2002; Murakami et al., 2002a, 2002b; Feklistov and Darst, 2011; Zhang et al., 2012). (**C**) Disposition of the W-dyad in RPo (containing upstream ds/ss junction, shown schematically above). Only the nt-strand DNA from −14 to −7, the $\sigma_{2\text{.}3}^{\text{A}}-\text{helix}$, and the W-dyad are shown (as in **B**). (**D**) Same view as (**C**). Superimposed is the simulated annealing omit map (grey mesh, 2*F*_o_ − *F*_c_, contoured at 1σ), calculated from a model where the following segments of σ^A^ were completely removed (216–221, 255–258, and 287–292) and shown only within 2 Å of omitted atoms. (**E**) Similar view as (**A**) (*right*). Superimposed is the simulated annealing omit map (grey mesh, 2*F*_o_ − *F*_c_, contoured at 1σ), calculated from a model where the following segments of σ^A^ were removed (216–221, 255–258, and 287–292) and shown only within 2 Å of omitted atoms. Clear Fourier density for σ^A^ Y217 and R288 is shown. **DOI:** http://dx.doi.org/10.7554/eLife.08504.011

Flipping of the A_−11_(nt) base from the duplex DNA into its recognition pocket in $\sigma_{2}^{\text{A}}$ is thought to be the key event in the initiation of promoter melting (Chen and Helmann, 1997; Lim et al., 2001; Heyduk et al., 2006; Feklistov and Darst, 2011). Strand opening propagates downstream to +1, but in the upstream direction, the base-paired T_−12_(nt) interacts with an invariant W-dyad of $\sigma_{2}^{\text{A}}$ (W256/W257, σ^70^ W433/W434; Figure 1—figure supplement 3; Table 3) to maintain the ds/ss (−12/−11) junction at the upstream edge of the transcription bubble (Figure 3A,C,D, Figure 3—figure supplement 1). The stabilization of the upstream ds/ss junction involves a previously unseen rearrangement of the W256 side chain. In all previous high resolution structures of σ^A^/σ^70^ in many different contexts but never with an upstream ds/ss junction (Malhotra et al., 1996; Campbell et al., 2002; Vassylyev et al., 2002; Feklistov and Darst, 2011; Zhang et al., 2012), the W256 side chain makes an ‘edge-on’ interaction with W257 (Figure 3B). In the presence of the upstream ds/ss junction, the W256 side chain rotates away from W257, filling the space vacated by the flipped-out A_−11_(nt) and forming a π-stack with the face of T_−12_(t) otherwise exposed by the absence of A_−11_(nt) (Figure 3C,D, Figure 3—figure supplement 1). The W-dyad forms a ‘chair’-like structure, with W256 serving as the back of the chair, and W257 as the seat, buttressing T_−12_(nt) from the major groove side (Figure 3A,C,D). The methyl group of the T_−12_(nt) base approaches the face of the W257 side chain at a nearly orthogonal angle, possibly forming a favorable methyl π interaction (Umezawa and Nishio, 1998; Brandl et al., 2001) (Figure 3C).

**Figure 3—figure supplement 1.**
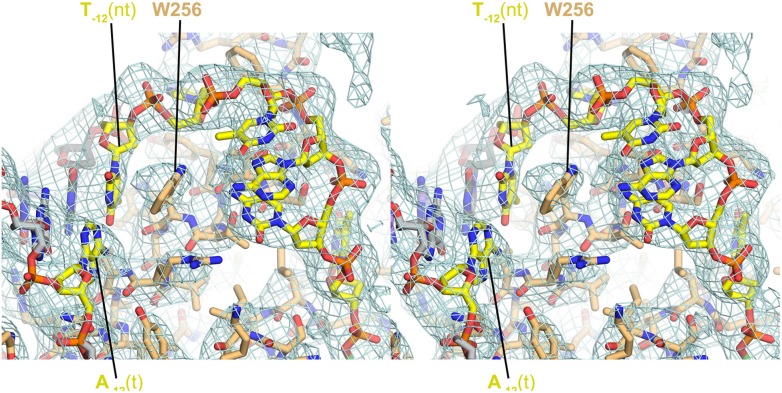
Stereo view of RPo model and resulting electron density map (grey mesh, 2*F*_*o*_ *− F*_*c*_, contoured at 0.7σ). The view is similar to Figure 3A. **DOI:** http://dx.doi.org/10.7554/eLife.08504.012

Examination of the structure near the upstream ds/ss junction revealed the solvent-exposed aromatic face of a conserved $\sigma_{2}^{\text{A}}$ Tyr side chain, Y217 (σ^70^ Y394; Figure 3A,E; Figure 1—figure supplement 3; Table 3), that does not appear to play an important role in the σ structure per se, but lies along the path the −11(t) base could follow from its position in duplex DNA (base-paired to A_−11_(nt)) to its position in the structure when orphaned by the flipped out A_−11_(nt) (dashed line, Figure 3A). The −11(t) nucleotide is almost always a T, being complementary to A_−11_(nt), the most highly conserved position of the −10 element (Shultzaberger et al., 2007). In the us-fork, the −11(t) nucleotide is absent (Figure 1—figure supplement 1), whereas in RPo, the −11(t) nucleotide is an (atypical) A, being part of the engineered non-complementary transcription bubble (Figure 1A). In RPo, the A_−11_(t) base is not stacked on Y217 but instead is about 12 Å away, flipped up alongside the $\sigma_{3}^{\text{A}}-3.0$ α-helix, sitting between R288 and R291 (Figure 3A; Figure 1—figure supplement 3; Table 3). We reasoned that we may not observe the orphaned −11(t) base stacked on Y217 for two reasons that are not mutually exclusive. First, Y217 may play an important role in stabilizing the melted state of the −11 bp during an intermediate of the normal promoter melting pathway (Saecker et al., 2011). Second, structural modeling suggested that the A_−11_(t) purine base present in the synthetic promoter construct (Figure 1A) may be too bulky to stack on Y217, which sits at the bottom of a narrow trough in the $\sigma_{2}^{\text{A}}$ structure (Figure 3A).

**Figure 4.**
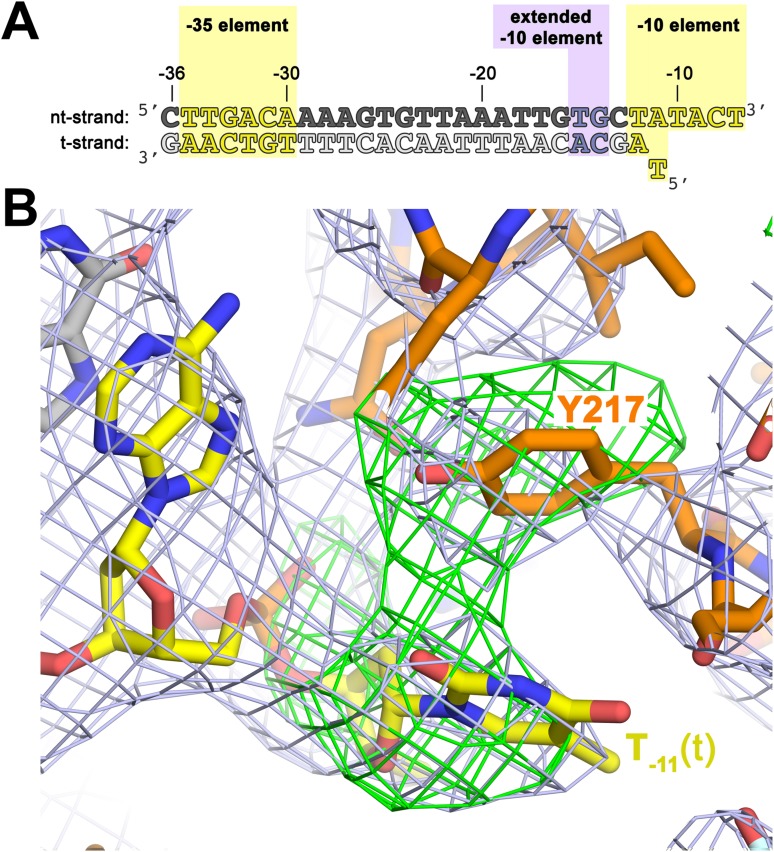
The σ^A^ Y217 may stack on the T_−11_(t) base orphaned by the flipped out A_−11_(nt) base. (**A**) Synthetic oligonucleotides used for us-fork (−11 bp) crystallization. The numbers above the sequence denote the DNA position with respect to the transcription start site (+1). The DNA sequence is derived from the full con promoter (Gaal et al., 2001). The −35 and −10 (Pribnow box) elements are shaded yellow, the extended −10 element (Keilty and Rosenberg, 1987) purple. The nt-strand DNA (top strand) is colored dark grey; the t-strand DNA (bottom strand), light grey; the RNA transcript, red. (**B**) The T_−11_(t) base orphaned by the flipped out A_−11_(nt) stacks on σ^A^ Y217 in the us-fork (−11 bp) structure. The 4.6 Å-resolution electron density map (contoured at 0.7σ) is shown (grey mesh). Also superimposed is the simulated annealing omit map (green mesh, *F*_o_ − *F*_c_, contoured at 3σ), calculated from a model where σ^A^ Y217 was mutated to Ala and the T_−11_(t) nucleotide was deleted. **DOI:** http://dx.doi.org/10.7554/eLife.08504.013

To investigate the role of Y217 further, we crystallized *Taq* EΔ1.1σ^A^ with an us-fork template containing a complementary A:T bp at the −11 position (us-fork (−11 bp); Figure 4A). To avoid model bias, we determined the structure by molecular replacement using the *Taq* EΔ1.1σ^A^/us-fork (−12 bp) structure (lacking the −11(t) base; Figure 1—figure supplement 1). The structure was modeled and refined (4.6 Å-resolution, Table 1, Figure 4—figure supplement 1), and the unbiased density maps revealed clear difference density for the T_−11_(t) base stacked on Y217 (Figure 4B).

**Figure 4—figure supplement 1.**
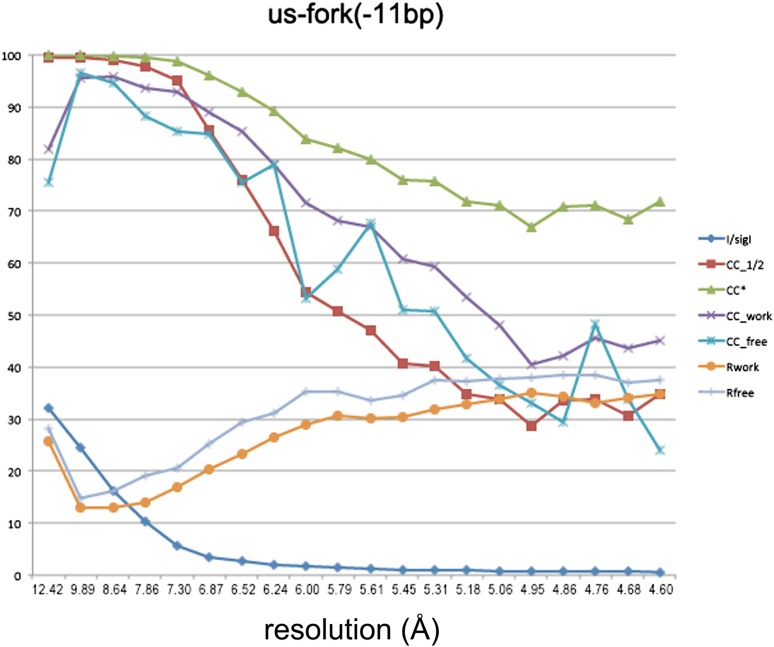
Data and model quality for us-fork (−11 bp) complex. Plots relating data quality with model quality at 4.6 Å-resolution) using the Pearson correlation coefficient (CC) analysis described by Karplus and Diederichs (2012). CC1/2 (red squares) was determined from the unmerged diffraction data randomly divided in half. Since CC1/2 underestimates the information content of the data (since it's calculated by dividing the dataset in half), CC* was calculated from an analytical relation to estimate the information content of the full data (Karplus and Diederichs, 2012). CC* provides a statistic that assesses data quality as well and also allows direct comparison of crystallographic model quality and data quality on the same scale through CC_work_ and CC_free_, the standard and cross-validated correlations of the experimental intensities with the intensities calculated from the refined model. A CC_work_/CC_free_ smaller than CC* indicates that the model does not account for all of the signal in the data, meaning it is not overfit. Plotted also are the standard <*I*>/σ*I* for the diffraction data, as well as the *R*_work_/*R*_free_ for the refined models. **DOI:** http://dx.doi.org/10.7554/eLife.08504.014

### Functional role of σ^A^ aromatic residues in forming and stabilizing the upstream ds/ss junction of the transcription bubble

A functional role for W256 in promoter melting was first proposed by Helmann and Chamberlin (1988). Ala substitution of the corresponding Trp in *Bacillus subtilis* σ^A^ gave rise to severe promoter melting defects in vitro and corresponding cold phenotypes in vivo (Juang and Helmann, 1994; Panaghie et al., 2000). The functional role of Y217 has not, to our knowledge, been previously examined.

We investigated the effects of individual Ala substitutions in *Eco* σ^70^ W433 and Y394 (*Taq* W256 and Y217) on the kinetics of RPo formation (Roe et al., 1984; Buc and McClure, 1985) using a recently reported fluorescence assay (Ko and Heyduk, 2014). The assay relies on a Cy3 fluorophore attached to the promoter nt-strand at position +2; fluorescence yield in this context is sensitive to the local environment and increases more than twofold upon RPo formation. Unlike previously used non-equilibrium methods (EMSA, filter binding), this assay allows detection of promoter melting at equilibrium and does not depend on the use of competitors, such as heparin. For these assays, we used one of the most thoroughly characterized promoters, λ P_R_ (Saecker et al., 2002, 2011). Control assays showed that under saturating conditions, both σ^70^ substitutions (W433A and Y394A) associated with core RNAP and supported abortive transcription as well as wild-type σ^70^ (data not shown), confirming their structural integrity.

**Figure 5.**
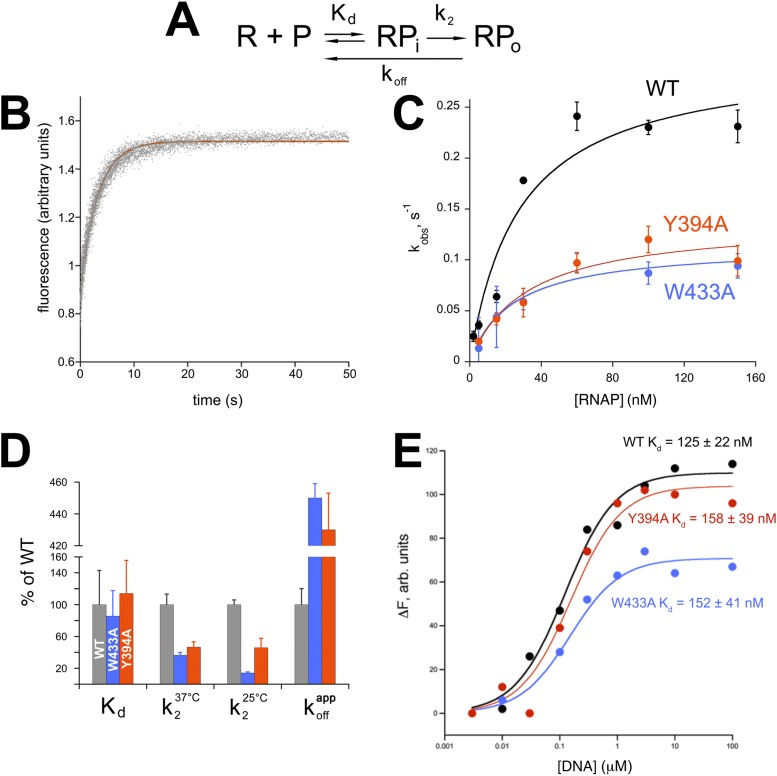
Functional role of *Eco* σ^70^ W433 and Y394 in RPo formation. (**A**) Simplified, two-step kinetic scheme for RPo formation (Roe et al., 1984; Buc and McClure, 1985) (R, RNAP; P, promoter; RP_i_, intermediate complex). (**B**) Representative time trace of fluorescence increase (from Cy3 labelled promoter DNA) during RPo formation. The solid red line illustrates the non-linear regression fit to a single-exponential model (see ‘Materials and methods’), which described >90% of the fluorescence amplitude rise. (**C**) The RNAP-concentration dependence of the observed rate (*k*_obs_) of RPo formation detected by Cy3 fluorescence (Ko and Heyduk, 2014) for *Eco* holoenzymes with σ^70^ (wt) as well as σ^70^ carrying substitutions W433A or Y394A. Error bars denote standard errors of the mean for ≥three independent measurements. (**D**) Summary of effects of σ^70^ W433A and Y394A substitutions on thermodynamic and kinetic parameters of RPo formation. The data was normalized to the % observed with wild-type Eσ^70^. (**E**) Equilibrium binding of ss nt-strand oligos of λ P_R_ promoter −10 element detected in the fluorescent RNAP beacon assay (Feklistov and Darst, 2011; Mekler et al., 2011) to *Eco* holoenzymes with σ^70^, as well as σ^70^ carrying substitutions W433A or Y394A. **DOI:** http://dx.doi.org/10.7554/eLife.08504.015

The multistep process of promoter opening can be described by a simplified kinetic scheme (Figure 5A) (McClure, 1980) where an initial promoter complex (RP_i_) existing in rapid equilibrium with free promoter and RNAP (binding step described by a dissociation constant *K*_d_) is converted in a rate-limiting step to RPo (isomerization described by the rate constant *k*_2_). Fluorescence traces of RPo formation under pseudo first-order conditions (Roe et al., 1984) recorded at increasing RNAP concentrations were fit to single-exponentials and yielded observed rate constants (*k*_obs_) for RPo formation (Figure 5B). Nonlinear fits to the resulting hyperbolic curves (Figure 5C) allowed the determination of *K*_d_ and *k*_2_ (Saecker et al., 2002) (Figure 5D).

**Figure 5—figure supplement 1.**
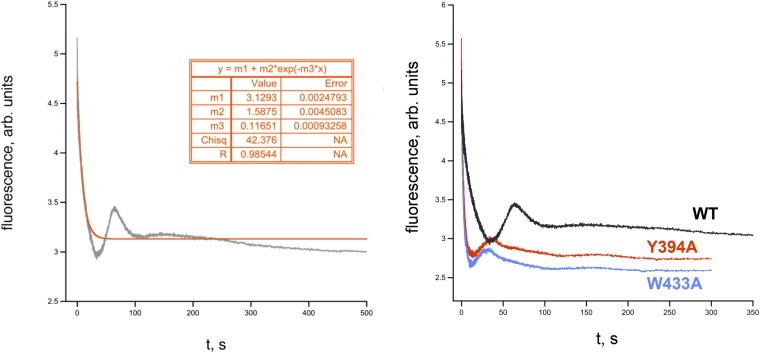
RPo dissociation data. (*Left*) Representative time trace of fluorescence decay after rapid mixing of pre-formed *Eco* RPo (with wild-type σ^70^) into 1.1 M NaCl (Gries et al., 2010). The solid line illustrates the non-linear regression fit to a single-exponential model. (*Right*) Representative dissociation curves for holoenzymes containing wild-type σ^70^ and W433A and Y394A substitutions. **DOI:** http://dx.doi.org/10.7554/eLife.08504.016

Neither σ^70^ W433A nor Y394A had a significant effect on *K*_d_ for RP_i_ formation, but the substitutions decreased the rate of isomerization by about twofold to threefold (at 37°C, Figure 5D). At suboptimal temperature (25°C) the effect of the W433A substitution was more pronounced, resulting in an ∼sevenfold reduction in isomerization rate. Neither σ^70^ W433A nor Y394A significantly altered the affinity of holoenzyme binding to ss oligos comprising the nt-strand of the −10 element (Tomsic, 2001) (Figure 5E).

W256 appears to make the primary contribution to maintaining the ds/ss junction at the upstream edge of the transcription bubble (Figure 3A), suggesting that this residue may play an important role in preventing transcription bubble collapse and dissociation of RPo. To probe the roles of both σ^70^ W433 and Y394 in maintaining RPo stability, we rapidly destabilized preformed RPo with 1.1 M NaCl (Gries et al., 2010) and followed the loss of RPo by monitoring the decay of fluorescence intensity with time (Figure 5—figure supplement 1). The dissociation curves are complex, reflecting the detection of a short lived intermediate (expected under these conditions) (Gries et al., 2010) by this assay. Although a full analysis is beyond the scope of this study, the overall apparent rate of RPo decay $\left(k_{\text{off}}^{\text{app}}\right)$ was determined from single-exponential fits of the decay curves. The σ^70^ W433A and the Y394A variants both gave a ∼fourfold higher rate of RPo dissociation under high salt conditions than did wild-type σ^70^ (Figure 5D, Figure 5—figure supplement 1).

### σ^A^ directs the ss t-strand to the RNAP active site

Downstream from the point of melting, the two DNA strands are directed on orthogonal paths (black arrows, Figure 3A). The nt-strand (−11 to −4) drapes across the surface of $\sigma_{2}^{\text{A}}$, directed by phosphate backbone interactions and notable base-specific recognition of A_−11_(nt) and T_−7_(nt) of the −10 element, and G_−6_(nt) of the discriminator (Feklistov and Darst, 2011; Zhang et al., 2012). Further downstream, interactions of the nt-strand from −3 to +2 occur exclusively with the RNAP β subunit, including base-specific recognition of G_+2_(nt) (Zhang et al., 2012).

**Figure 6.**
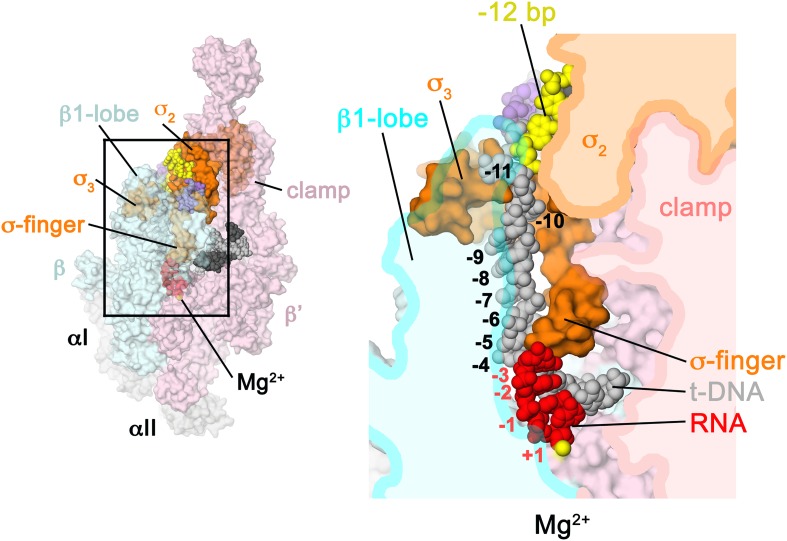
Structural role of the σ_3.2_-loop. (*Left*) Overall view of RPo structure, colored as in Figure 1 except σ^A^ is orange. The RNAP β and β′ subunits are transparent to reveal the RNAP active site Mg^2+^ (yellow sphere) and the nucleic acids held inside the RNAP active site channel. The ss nt-strand DNA is omitted for clarity. The boxed area is magnified on the right. (*Right*) Magnified view showing a cross-section of the RNAP active site channel. For clarity, the RNAP β, β′, and $\sigma_{2}^{\text{A}}$ domains are shown mostly as outlined shapes, with β transparent. The ss t-strand DNA (−11 to −4) is directed towards the RNAP active site through a tunnel between the σ_3.2_-loop and the β1-lobe. The 4-nt RNA transcript (−3 to +1) contacts the distal tip of the σ_3.2_-loop. Further elongation of the RNA would require displacement of the σ_3.2_-loop. **DOI:** http://dx.doi.org/10.7554/eLife.08504.017

At the point of melting, a ∼90° turn of the t-strand backbone (between −12 and −11) may be effected by electrostatic interactions between conserved basic residues of $\sigma_{2}^{\text{A}}$ (R220; Figure 1—figure supplement 3; Table 3) and $\sigma_{3}^{\text{A}}$ (R288, R291) and four t-strand backbone phosphates in a row (−13, −12, −11, −10) encompassing the turn (Figure 3A). Strong simulated annealing omit 2*F*_o_ − *F*_c_ density is associated wth $\sigma_{3}^{\text{A}}$ R288, confirming its role in interacting with the −13(t) phosphate (Figure 3E). The $\sigma_{2}^{\text{A}}$ R220 and $\sigma_{3}^{\text{A}}$ R291 give weaker difference density so their role in interacting with the −12(t) and −11(t) phosphate groups is tentative. The turn directs the t-strand away from the nt-strand and towards the RNAP active site (Figure 3A). The ss t-strand DNA from −9 to −5 is guided towards the RNAP active site through a tunnel formed between the RNAP β1-lobe (called the protrusion in eukaryotic RNAP II; Cramer et al., 2001) and the σ_3.2_-loop (also referred to as the σ-finger), an extended linker that loops into and out of the RNAP active-site channel (Murakami et al., 2002a; Zhang et al., 2012), connecting the σ_3_ and σ_4_ domains (Figure 6).

### The σ_3.2_-loop sterically blocks extension of the 4 nt RNA transcript

Previous structural analyses predicted that the σ_3.2_-loop would physically occupy the path of the elongating RNA and must be displaced for full RNA extension to occur (Vassylyev et al., 2002; Murakami et al., 2002a). Indeed, the upstream edge of the post-translocated 4-nt transcript fits snugly between the RNAP active site and the distal tip of the σ_3.2_-loop, which contacts the upstream RNA:DNA bp at −3, and the t-strand bases at −4 and −5 (Figure 6). Extension of the RNA transcript and translocation to form a 5 bp post-translocated RNA:DNA hybrid cannot occur without displacement of the σ_3.2_-loop (Basu et al., 2014), marking the point in transcription initiation (translocation of the 4–5 bp RNA:DNA hybrid from pre- to post-translocated) where steric clash between the elongating RNA transcript and the σ_3.2_-loop begins effecting abortive initiation and σ release (Murakami et al., 2002a; Nickels et al., 2005; Kulbachinskiy and Mustaev, 2006).

## Discussion

Our structures reveal that the overall architecture of the *Taq* RPo (Figure 1) closely resembles that of the *Eco* RPo (Zuo and Steitz, 2015), but the improved resolution of our analysis allows a more detailed description of protein/DNA interactions (Figure 2), particularly interactions involved in forming and stabilizing the ds/ss junction at the upstream edge of the transcription bubble (Figure 3). Previous models of RPo were pieced together from structures of σ domains or RNAP holoenzyme complexed with promoter fragments (Campbell et al., 2002; Murakami et al., 2002b; Feklistov and Darst, 2011; Zhang et al., 2012). The *Taq* RPo structure upstream of the −10 element matches the overall architecture of the low-resolution (6.5 Å) *Taq* RNAP holoenzyme/upstream-fork promoter complex (Murakami et al., 2002b) except unlike the upstream-fork structure (where the RNAP holoenzyme/−35 element interactions were distorted by crystal packing interactions), the *Taq* RPo recapitulates the σ_4_/−35 element interactions seen in the high-resolution (2.4 Å) crystal structure of the *Taq* $\sigma_{4}^{\text{A}}/-35$ element DNA complex (Campbell et al., 2002). The *Taq* RPo structure also recapitulates the σ_2_/−10 element interactions seen in high-resolution (2.1 Å) structures of *Taq* $\sigma_{2}^{\text{A}}$ complexes with ss −10 element DNA (Feklistov and Darst, 2011). The interactions of the RNAP holoenzyme with the ss discriminator element (ss nt-strand DNA from −6 to −3; Figure 1A), the ss nt-strand DNA from −2 to +2 (including base-specific interactions of G_+2_(nt) with a pocket in the RNAP β subunit), and the downstream edge of the transcription bubble and downstream duplex DNA are very similar to those observed in a 2.9 Å-resolution structure of *Tth* RNAP holoenzyme complexed with a downstream-fork promoter template (Zhang et al., 2012).

### Role of conserved σ^A^ aromatic residues in promoter opening

Our results clarify the role of the universally conserved W-dyad of housekeeping (also called primary or group 1) σ's (Gruber and Bryant, 1997) in the promoter opening pathway, particularly for *Taq* σ^A^ W256 (*Eco* σ^70^ W433), which rotates into the DNA duplex and serves as a steric mimic of the flipped-out A_−11_(nt) base by a stacking mechanism (Figure 3A,C,D). The bacterial RNAP σ subunit can be added to the list of proteins using a wedge residue (usually an aromatic side chain) to invade the DNA duplex to stabilize the extrahelical conformation of a flipped-out base (Lau et al., 1998; Davies et al., 2000; Yang et al., 2009; Yi et al., 2012). We also identified another conserved σ^A^ aromatic residue (*Taq* σ^A^ Y217) that plays an important role in the promoter opening pathway, possibly by stacking with T_−11_(t) orphaned when the conserved A_−11_(nt) base flips out (Figure 4B).

The kinetic studies reveal that both aromatic residues (W256 and Y217) act in a context dependent manner—they are not important for the initial promoter binding step (Figure 5D) nor for binding the ss −10 element DNA (Figure 5E): instead W256 and Y217 act to increase the rate of the isomerization (promoter opening step) itself (Figure 5E,D), possibly by making contacts unique to the transition state that lower the energy barrier between RPi and RPo in the two-step kinetic scheme (Figure 5A). Since the initial promoter binding step (formation of RPi, Figure 5A) is not affected by the σ^70^ W433A substitution (Figure 5D), we surmise that RPi does not feature the stacking interaction formed by W433A on the T_−12_(nt) base (exposed by the flipping-out of A_−11_(nt)). Since the −11 bp is thought to be the first bp disrupted in the promoter opening pathway (Chen and Helmann, 1997; Lim et al., 2001; Heyduk et al., 2006; Feklistov and Darst, 2011), this implies that RPi is a closed complex (RPc) comprising duplex promoter DNA.

The effects of σ^70^ W433A that we observed are consistent with previous observations using nonequilibrium methods (Fenton et al., 2000; Tomsic, 2001; Fenton and Gralla, 2003; Schroeder et al., 2009). These observations support the critical role of σ^A^ W256 and Y217 (σ^70^ W433 and Y394) in formation and stability of RPo.

In addition to the housekeeping σ (σ^A^ in *Taq* or σ^70^ in *Eco*) that controls transcription of the majority of cellular genes (with consensus −35 and −10 elements of TTGACA and TATAAT, respectively; Shultzaberger et al., 2007), bacteria rely on alternative σ′s to direct RNAP to highly specialized promoters (with alternative −35 and −10 elements) controlling operons in response to environmental and physiological cues (Gruber and Gross, 2003; Feklistov et al., 2014). Although the W-dyad is universally conserved in housekeeping σ's (Gruber and Bryant, 1997), it is not a conserved feature of alternative σ's (Lonetto et al., 1992; Helmann, 2002; Campbell et al., 2003); bulky hydrophobic residues are favored at the corresponding positions of alternative σ's (but rarely W). The W-dyad is likely to be the optimal configuration for supporting the upstream ds/ss junction of the transcription bubble, giving the housekeeping σ′s a powerful DNA-melting capacity, allowing them to function on thousands of highly divergent, nonoptimal promoter sequences. Alternative residues supporting the upstream ds/ss junction of the transcription bubble may weaken the ability of RNAP with alternative σ's to form RPo, fine-tuning their specificity (Feklistov et al., 2014). The residue corresponding to *Taq* σ^A^ Y217 (σ^70^ Y394) appears to be conserved as either Y or F among σ^70^-family alternative σ's suggesting that this residue plays a key role common to all σ′s.

### Transcript elongation, scrunching, and σ-release

Zuo and Steitz (2015) soaked crystals of *Eco* transcription initiation complexes (containing a full transcription bubble) with NTP substrates to generate short transcripts (with 5′-triphosphate) *in crystallo*. A pre-translocated 4-nt transcript did not reach the σ_3.2_-loop, whereas a pre-translocated 5-nt transcript appeared to just reach and interact with the σ_3.2_-loop. Attempts to generate longer transcripts resulted in severe degradation of the crystals, suggesting significant conformational changes of the RNAP that were incompatible with the crystal packing either due to transcript/σ_3.2_-loop interactions, ‘scrunching’ of the t-strand DNA (Kapanidis et al., 2006; Revyakin et al., 2006; Roberts, 2006), or both. The upstream edge of our post-translocated 4-nt transcript is equivalent to the pre-translocated 5-nt transcript observed by Zuo and Steitz (2015): in both cases the upstream edge of the RNA just contacts the σ_3.2_-loop and the conformation of the σ_3.2_-loop is very similar indicating that, at least in this case, the presence or absence of the 5′-triphosphate does not alter the gross interaction of the elongation transcript with the σ_3.2_-loop. In vitro, RNAP initiates efficiently with dinucleotide primers lacking a 5′-triphosphate without obvious defects in σ release or promoter escape.

Basu et al. (2014) were able to generate a 6-nt pre-translocated transcript (containing a 5′-triphosphate) in crystals of *Tth* transcription initiation complexes with a downstream-fork promoter template that lacks duplex DNA upstream of the −10 element and is therefore unable to ‘scrunch’ the t-strand DNA. In this case, the 5′-nt of the transcript displaces the σ_3.2_-loop, which is not modeled and presumably disordered. Other conformational changes of the RNAP or changes in σ/RNAP interactions were not observed.

### Relationship to RPo formation in eukaryotes

In vitro, the rate-limiting step of bacterial RNAP transcription is often the isomerization step to open the promoter and form RPo (McClure, 1980, 1985; Amouyal and Buc, 1987). The kinetics of the many steps of the transcription cycle in vivo have not been characterized, but many transcription units are clearly controlled at the initiation step (Paul et al., 2004). In bacteria, recognition of the promoter −10 element and DNA opening are directly coupled (Feklistov and Darst, 2011; Liu et al., 2011), with the Trp stacking interaction (Figure 3A,C) playing a key role.

In contrast to tight coupling between promoter recognition and transcription bubble formation at most bacterial promoters, in eukaryotes promoter recognition, RNAP II recruitment, and promoter opening appear to be uncoupled. The preinitiation complex (PIC) is the molecular assembly through which eukaryotic RNAP II locates and utilizes a promoter, which may be pre-recognized by basal transcription factors. RPo formation requires ATP hydrolysis by the Ssl2 (XPB) subunit of TFIIH, which translocates downstream DNA into RNAP II against fixed upstream contacts to force DNA melting (Kim et al., 2000; Grünberg and Hahn, 2013). This contrasts with the spontaneous unwinding driven by RNAP/promoter DNA interactions alone during bacterial RPo formation (Liu et al., 2011).

Although there are clear similarities between σ and the eukaryotic basal transcription factor IIB in the contacts made to the 5′ RNA, hybrid junction, and ss-tDNA, there is no structural similarity between σ and TFIIB (Kostrewa et al., 2010; Liu et al., 2010; Sainsbury et al., 2013). These contacts may play similar roles in aiding promoter escape by helping eject σ or TFIIB from the RNAP active site cleft, but it is currently unclear whether any eukaryotic basal transcription factor stabilizes an upstream fork-junction by interactions similar to the σ-mediated Trp stacking (Figure 3A,C). Further, although effects on RPo formation may help regulate some eukaryotic promoters (Kouzine et al., 2013), other steps, including removal of nucleosomes and promoter-proximal pausing (Boeger et al., 2003; Adelman and Lis, 2012) appear to be rate-limiting at many eukaryotic promoters. Even when promoters are nucleosome-free, assembly of the PIC, rather than promoter opening, may be rate-limiting. Further mechanistic and structural studies of RNAPII on promoters with diverse architectures, including both TATA-containing and TATA-less promoters, are needed for a better understanding of the steps in RNAPII initiation.

### Conclusions

The structures of RPo determined here reveal how the RNAP holoenzyme recognizes the extended −10 element, stabilizes the transcription bubble, directs the t-strand DNA into the RNAP active site, and how the RNA:DNA hybrid initiates σ^A^ release. Supported by the real-time kinetic data, the structures elucidate the roles of individual aromatic amino acid residues in nucleation of the transcription bubble and maintenance of RPo stability, in part through previously unobserved stacking mechanisms. The results also provide a basis for more incisive investigations of RPo formation and transcriptional regulation (Bae et al., 2015).

## Materials and methods

### Preparation and crystallization of *Taq* Δ1.1σ^A^-holoenzyme/promoter complexes

*Taq* core RNAP and Δ1.1σ^A^ were prepared as described previously (Murakami et al., 2003). Promoter DNA strands (Oligos Etc.) were annealed in 10 mM Tris–HCl, pH 8.0, 1 mM EDTA, 0.2 M NaCl and aliquots were stored at −20°C.

For crystallization, aliquots of purified *Taq* core RNAP and Δ1.1σ^A^ were thawed on ice and buffer-exchanged into crystallization buffer (20 mM Tris–HCl, pH 8.0, 0.2 M NaCl). *Taq* Δ1.1σ^A^-holoenzyme was formed by adding 1.2-fold molar excess of Δ1.1σ^A^ to the core RNAP and the mixture was incubated for 15 min at room temperature. A 1.5-fold molar excess of promoter DNA was then added to the holoenzyme along with MgCl_2_ (10 mM final) and incubated for 15 min at room temperature. When present, a fivefold molar excess of RNA primer (GE Dharmacon, Lafayette, CO, United States) was also added. The final RNAP concentration was adjusted to 25 μM. Crystals were grown by vapor diffusion at 22°C by mixing 1 μl of sample with 1 μl of reservoir solution (20 mM MgCl_2_, 20 mM Tris–HCl, pH 8.0, 1.6 M ammonium sulfate) in a 48-well hanging drop tray (Hampton Research, Aliso Viejo, CA, United States). Thin rod-shaped crystals (typically, 30 × 30 × 300 μm) appeared after about 5 days. The crystals were transferred into reservoir solution supplemented with 25% (vol/vol) glycerol in two steps for cryo-protection, then flash frozen by plunging into liquid nitrogen.

### Structure determination

X-ray diffraction data were collected at Brookhaven National Laboratory National Synchrotron Light Source (NSLS) beamline X29 and at Argonne National Laboratory Advanced Photon Source (APS) NE-CAT beamlines 24-ID-C and 24-ID-E. Data were integrated and scaled using HKL2000 (Otwinowski and Minor, 1997). The diffraction data were anisotropic. To compensate, isotropy was approximated by applying a positive b factor along a* and b* and a negative b factor along c* (Table 1), as implemented by the UCLA MBI Diffraction Anisotropy Server (http://services.mbi.ucla.edu/anisoscale/) (Strong et al., 2006), resulting in enhanced map features (Figure 1C, Figure 1—figure supplements 1, 2C,D, 3D,E, Figure 3—figure supplement 1, Figure 4B).

Initial electron density maps were calculated by molecular replacement using Phaser (McCoy et al., 2007) from a starting model of *Taq* Δ1.1σ^A^-holoenzyme determined at 2.8 Å-resolution (unpublished). Two RNAP/DNA complexes were clearly identified in the asymmetric units. The models were first improved using rigid body refinement of each RNAP molecule and subsequently of 20 individual mobile domains using PHENIX (Adams et al., 2010). At this point, the electron density maps showed strong connected difference density for the nucleic acids, allowing unambiguous placement using COOT (Emsley and Cowtan, 2004). Detailed nucleic acid modeling was facilitated using available models of complexes with promoter fragments: $\sigma_{4}^{\text{A}}/-35$ element DNA complex at 2.4 Å (1KU7 [Campbell et al., 2002]), RNAP-holoenzyme/us-fork DNA at 6.5 Å-resolution (1L9Z [Murakami et al., 2002b]), $\sigma_{2}^{\text{A}}/\text{nt}-\text{strand}$ −10 element DNA at 2.1 Å (3UGO [Feklistov and Darst, 2011]), RNAP-holoenzyme/downstream-fork DNA at 2.9 Å (4G7H [Zhang et al., 2012]), RNA/DNA hybrid at 2.5 Å (2O5I [Vassylyev et al., 2007]). The resulting models were improved using deformable elastic network (DEN) refinement (Schröder et al., 2010) with noncrystallographic symmetry (NCS) restraints using CNS 1.3 (Brunger et al., 1998) performed on the Structural Biology Grid portal (O'Donovan et al., 2012), followed by iterative cycles of manual building with COOT (Emsley and Cowtan, 2004) and refinement with PHENIX (Adams et al., 2010).

In the RPo structure, the ss t-strand DNA from −11 to −4 was only modeled in one complex of the asymmetric unit. In the other complex, strong, connected Fourier difference density for this segment of DNA was observed but the density was relatively featureless and we were unable to model this segment of the DNA. In the us-fork (−11 bp) complex, the t-strand T_−11_ was modeled in only one complex of the asymmetric unit. In the other complex, density for this base was absent.

### Resolution limit and structure validation

We follow the criteria of Karplus and Diederichs (2012), who showed that the *R*_merge_ statistic commonly used to evaluate data quality is ‘seriously flawed’ and should not be used (Diederichs and Karplus, 1997), and that the commonly used criteria of <*I*>/σ*I* > 2 also results in the loss of much useful crystallographic data (Karplus and Diederichs, 2012). Karplus and Diederichs (2012) showed, using objective and unbiased analyses, that inclusion of weak X-ray diffraction data (*R*_merge_ values >> 1.0 and <*I*>/σ*I* << 1) resulted in improved structural models. An improved statistic, CC* (essentially a Pearson correlation coefficient), was introduced that provides a single statistically valid guide for deciding whether diffraction data are useful.

Since most of the analyses described herein were performed from the RPo structure, we justify the inclusion of diffraction data to 4.14 Å-resolution for this case. Data in the highest resolution shell (4.29–4.14 Å) are very weak when examined by standard criteria (high *R*_pim_ values and <*I*>/σ*I* = 0.8, Table 1), but have good multiplicity (21.6) and completeness (99.8%), and yield a CC1/2 of 0.157, which is significantly different from zero for the large sample size (16,966 unique reflections) at exceedingly low p values (Rahman, 1968). That the highest resolution shells contain useful data and not noise is reflected in the observation that the *R*_free_ and *R*_work_ for the model refinement do not diverge (Figure 1—figure supplement 2, Figure 4—figure supplement 1). Inclusion of higher resolution data resulted in unacceptably low completeness in the highest shells due to the data anisotropy.

In the final 2*F*_o_ − *F*_c_ electron density maps, numerous protein side chains were resolved, including many that appeared to form important protein/nucleic acid interactions. To confirm these protein side chain positions, we produced unbiased difference Fourier maps using a simulated annealing omit procedure. Protein segments flanking the side chains in question were removed completely from the structural model, and the modified models were subjected to simulated annealing refinement using PHENIX (Adams et al., 2010). We used the following annealing temperatures (K), 1000; 2500; 5000; 10,000. All temperatures gave the same result (recovery of electron density for the omitted side chains), but the 5000 and 10,000 K refinements gave rise to obvious local structural distortions (expected for such high annealing temperatures with our low-resolution data) so the unbiased 2*F*_o_ − *F*_c_ maps were calculated from the 2500 K annealing refinements (Figures 2C,D, 3D,E).

### Kinetic measurements

#### Preparation of *Eco* core RNAP, **σ**^70^, and **σ**^70^ mutants

*Eco* core RNAP was overexpressed and purified from *Eco* BL21 (DE3) cells co-transformed with pGEMABC (encoding *Eco* RNAP *rpoA*, *rpoB*, and *rpoC*; Addgene plasmid 45398) and pACYCDuet-1_Ec_rpoZ (encoding *rpoZ*) as described (Murakami, 2013). *Eco* σ^70^ was overexpressed and purified as described previously (Feklistov and Darst, 2011). *Eco* σ^70^ W433A and Y394A substitutions were generated by site-directed mutagenesis of pGEMD-σ^70^ and purified using the same procedure as wild-type σ^70^.

#### Preparation of DNA for kinetic measurements

A 135 bp λ P_R_ promoter with Cy3 label at position +2 of the nontemplate strand was prepared using a 79 nt long synthetic oligonucleotide containing amino-dT at +2:

ATCTATCACCGCAAGGGATAAATATCTAACACCGTGCGTGTTGACTATTTTACCTCTGGCGGTGATAATGGTTGCA/iAmMC6T/GT

The oligonucleotide was modified with Cy3-NHS and purified by reverse phase HPLC. The duplex was then prepared by *Taq* DNA polymerase extension of a partial duplex formed by mixing 0.25 µM Cy3-labeled non-template strand and 0.275 µM 79 nt template strand (TGCTGACTGCTTAATCGCTTCTAGGGATATAGGTAATTCCATACCACCTCCTTACTACATGCAACCATTATCACCGCCA) containing at the 3′-end a 23 bp sequence complementary to the 3′-end of the nontemplate strand. Extended duplex was purified on a 1 ml Resource Q column (GE Healthcare Bio-Sciences, Marlborough, MA, United States) using a gradient of 0–1 M NaCl in 25 mM Tris–HCl (pH 8), 10 µM EDTA. Fractions containing labeled promoter were precipitated with ethanol to remove salt.

#### Mechanistic model

Quantitative mechanistic studies have found at least two kinetically significant intermediates (designated I_1_ and I_2_) on the pathway to formation of RPo by *Eco* RNAP at the λ P_R_ promoter (Davis et al., 2007; Gries et al., 2010; Saecker et al., 2011):

(1) $$\text{R}+\text{P}\overset{\begin{array}{c} \text{rapid}\\ \text{equilibrium} \end{array}}{\overset{k_{1}^{\prime }}{\underset{k_{-1}^{\prime }}{⇄}}}{\text{I}}_{1}\overset{\begin{array}{c} \text{slow}\\ k_{2}^{\prime } \end{array}}{\underset{\begin{array}{c} k_{-2}^{\prime }\\ \text{slow} \end{array}}{⇄}}{\text{I}}_{2}\underset{\begin{array}{c} \text{rapid}\\ \text{equilibrium} \end{array}}{\overset{k_{3}^{\prime }}{\underset{k_{-3}^{\prime }}{⇄}}}\text{RPo},$$

where the interconversion between I_1_ and I_2_ is rate-limiting in both directions (Buc and McClure, 1985; Saecker et al., 2002). The rate limiting step in the forward direction is the conversion of I_1_ to I_2_, so under standard solution conditions, I_2_ is never significantly populated (Gries et al., 2010). Because I_2_ is not significantly populated under the conditions of association experiments, the three-step mechanism simplifies to the two-step mechanism (Figure 5A), where I_1_ = RPi. Since the kinetics observed in the forward direction are well fit by a single exponential (Figure 5B), we deduce that RPi does not give rise to a significant fluorescence signal in our assay.

In the reverse direction, however, rapid destabilization of RPo (such as with 1.1 M NaCl used here) generates a burst of I_2_ (Kontur et al., 2006; Gries et al., 2010). The complexity and shapes of the dissociation curves observed by our fluorescence assay are consistent with the detection of a transient burst of I_2_ after challenging pre-formed RPo with 1.1 M NaCl (Figure 5—figure supplement 1) (Gries et al., 2010). Real-time observation of I_2_ is an important finding that merits further, quantitative study but is beyond the scope of this study. Instead, we have characterized the overall dissociation rate $\left(k_{\text{off}}^{\text{app}}\right)$ by fitting the dissociation curves with a single exponential, which reveals the gross (>fourfold) differences in overall dissociation rates observed between wild type and mutant σ's (Figure 5D, Figure 5—figure supplement 1).

#### Forward kinetics

To measure the kinetics of RPo formation, *Eco* RNAP holoenzyme was loaded in one syringe of a stopped-flow instrument (SF-300X, KinTek Corporation, Austin, TX, United States) and Cy3-labelled promoter DNA in the other. After rapid mixing at the indicated temperature (37°C or 25°C), the final concentrations were: promoter DNA, 0.3 nM; RNAP, 2 to 150 nM in binding buffer (20 mM HEPES, pH 8.0, 100 mM K-Glutamate, 10 mM MgCl_2_, 1 mM DTT). Cy3 fluorescence emission was measured in real time with a 586/20 single-band bandpass filter (Semrock) and excitation at 550 nm. The kinetics of Cy3 fluorescence were determined at various RNAP concentrations and fit to a single exponential equation (Figure 5B):

(2) $$F_{t}=F_{\infty }+\left(F_{0}-F_{\infty }\right){\text{e}}^{-k_{\text{obs}}t},$$

where *F*_*t*_ is the fluorescence intensity of Cy3 as a function of time (*t*), *F*_0_ is the initial fluorescence intensity, *F*_∞_ is the fluorescence intensity at *t* = ∞, and *k*_obs_ is the pseudo-first-order observed rate constant of the increase in Cy3 fluorescence. The data were interpreted assuming the following kinetic scheme (Figure 5A; [McClure, 1980; Buc and McClure, 1985]):

(3) $$\text{R}+\text{P}\overset{K_{\text{d}}}{⇄}\text{RPi}\overset{k_{2}}{\to }\text{RPo},$$

where the initial RNAP/promoter complex (RP_i_) existing in rapid equilibrium with free promoter and RNAP (described by a dissociation equilibrium constant *K*_d_) is converted in a rate-limiting step to RPo (described by the rate constant *k*_2_). To obtain *K*_d_ and *k*_2_ the observed rate constants (*k*_obs_, average values determined from >3 replicates) were plotted against RNAP concentrations (Figure 5C) and the data were fit to a hyperbolic equation (Saecker et al., 2002):

(4) $$k_{\text{obs}}=\frac{k_{2}\left[\text{RNAP}\right]}{\left[\text{RNAP}\right]+K_{\text{d}}}.$$

#### Reverse kinetics

Cy3-labeled DNA promoter fragments (0.3 nM) in binding buffer were mixed with RNAP-holoenzyme (100 nM) and incubated at 37°C for 20 min to preform RPo. They were rapidly mixed in the stopped-flow instument with the same buffer but resulting in a final NaCl concentration of 1.1 M. The kinetics of high-salt induced RPo decay was recorded in the same manner as for the forward direction. Averaged time traces from ≥3 replicates were fit to a single exponential Equation 2 corresponding to a simplified kinetic scheme:

(5) $$\text{RPo}\overset{k_{\text{off}}^{\text{app}}}{\to }\text{R}+\text{P}.$$

#### Accession numbers

The structure factor files and X-ray crystallographic coordinates have been deposited in the Protein Data Bank under ID codes 4XLP (*Taq* holoenzyme/us-fork (−12 bp) complex), 4XLQ (*Taq* holoenzyme/us-fork (−11 bp) complex), and 4XLN (*Taq* RPo).

## Additional files

### Major datasets

The following datasets were generated:

Bae B, Darst SA, 2015, Crystal structure of T.aquaticus transcription initiation complex containing upstream fork promoter, http://www.rcsb.org/pdb/search/structidSearch.do?structureId=4XLP, Publicly available at the RCSB Protein Data Bank (Accession no: 4XLP).

Bae B, Darst SA, 2015, Crystal structure of T.aquaticus transcription initiation complex containing upstream fork (−11 base-paired) promoter, http://www.rcsb.org/pdb/search/structidSearch.do?structureId=4XLQ, Publicly available at the RCSB Protein Data Bank (Accession no: 4XLQ).

Bae B, Darst SA, 2015, Crystal structure of T. aquaticus transcription initiation complex containing bubble promoter and RNA, http://www.rcsb.org/pdb/search/structidSearch.do?structureId=4XLN, Publicly available at the RCSB Protein Data Bank (Accession no: 4XLN).

## References

[bib1] Adams PD, Afonine PV, Bunkóczi G, Chen VB, Davis IW, Echols N, Headd JJ, Hung L-W, Kapral GJ, Grosse-Kunstleve RW, McCoy AJ, Moriarty NW, Oeffner R, Read RJ, Richardson DC, Richardson JS, Terwilliger TC, Zwart PH (2010). PHENIX: a comprehensive Python-based system for macromolecular structure solution. Acta Crystallographica. Section D, Biological Crystallography.

[bib2] Adelman K, Lis JT (2012). Promoter-proximal pausing of RNA polymerase II: emerging roles in metazoans. Nature Reviews Genetics.

[bib3] Amouyal M, Buc H (1987). Topological unwinding of strong and weak promoters by RNA polymerase. A comparison between the lac wild-type and the UV5 sites of *Escherichia coli*. Journal of Molecular Biology.

[bib4] Bae B, Chen J, Davis E, Leon K, Darst SA, Campbell EA (2015). CarD uses a minor groove wedge mechanism to stabilize the RNA polymerase open promoter complex. eLife.

[bib8] Barne KA, Bown JA, Busby SJW, Minchin SD (1997). Region 2.5 of the *Escherichia coli* RNA polymerase sigma70 subunit is responsible for the recognition of the ‘extended-10’ motif at promoters. The EMBO Journal.

[bib9] Basu RS, Warner BA, Molodtsov V, Pupov D, Esyunina D, Fernández-Tornero C, Kulbachinskiy A, Murakami KS (2014). Structural basis of transcription initiation by bacterial RNA polymerase holoenzyme. The Journal of Biological Chemistry.

[bib10] Boeger H, Griesenbeck J, Strattan JS, Kornberg RD (2003). Nucleosomes unfold completely at a transcriptionally active promoter. Molecular Cell.

[bib11] Brandl M, Weiss MS, Jabs A, Sühnel J, Hilgenfeld R (2001). C-H...pi-interactions in proteins. Journal of Molecular Biology.

[bib12] Brunger AT, Adams PD, Clore GM (1998). Crystallography & NMR system: a new software suite for macromolecular structure determination. Acta Crystallographica. Section D, Biological Crystallography.

[bib13] Buc H, McClure WR (1985). Kinetics of open complex formation between *Escherichia coli* RNA polymerase and the lac UV5 promoter. Evidence for a sequential mechanism involving three steps. Biochemistry.

[bib14] Campbell EA, Muzzin O, Chlenov M, Sun JL, Olson CA, Weinman O, Trester-Zedlitz ML, Darst SA (2002). Structure of the bacterial RNA polymerase promoter specificity sigma subunit. Molecular Cell.

[bib15] Campbell EA, Tupy JL, Gruber TM, Wang S, Sharp MM, Gross CA, Darst SA (2003). Crystal structure of *Escherichia coli* sigmaE with the cytoplasmic domain of its anti-sigma RseA. Molecular Cell.

[bib16] Chen YF, Helmann JD (1997). DNA-melting at the *Bacillus subtilis* flagellin promoter nucleates near -10 and expands unidirectionally. Journal of Molecular Biology.

[bib17] Cramer P, Bushnell DA, Kornberg RD (2001). Structural basis of transcription: RNA polymerase II at 2.8 Ångstrom resolution. Science.

[bib18] Daniels D, Zuber P, Losick R (1990). Two amino acids in an RNA polymerase sigma factor involved in the recognition of adjacent base pairs in the −10 region of a cognate promoter. Proceedings of the National Academy of Sciences of the USA.

[bib19] Davies DR, Goryshin IY, Reznikoff WS, Rayment I (2000). Three-dimensional structure of the Tn5 synaptic complex transposition intermediate. Science.

[bib20] Davis CA, Bingman CA, Landick R, Record MT, Saecker RM (2007). Real-time footprinting of DNA in the first kinetically significant intermediate in open complex formation by *Escherichia coli* RNA polymerase. Proceedings of the National Academy of Sciences of the USA.

[bib21] Diederichs K, Karplus PA (1997). Improved R-factors for diffraction data analysis in macromolecular crystallography. Nature Structural Biology.

[bib22] Emsley P, Cowtan K (2004). Coot: model-building tools for molecular graphics. Acta Crystallographica. Section D, Biological Crystallography.

[bib23] Feklistov A, Barinova N, Sevostyanova A, Heyduk E, Bass I, Vvedenskaya I, Kuznedelov K, Merkienė E, Stavrovskaya E, Klimašauskas S, Nikiforov V, Heyduk T, Severinov K, Kulbachinskiy A (2006). A basal promoter element recognized by free RNA polymerase σ subunit determines promoter recognition by RNA polymerase holoenzyme. Molecular Cell.

[bib24] Feklistov A, Darst SA (2011). Structural basis for promoter -10 element recognition by the bacterial RNA polymerase σ subunit. Cell.

[bib25] Feklistov A, Sharon BD, Darst SA, Gross CA (2014). Bacterial sigma factors: a historical, structural, and genomic perspective. Annual Review of Microbiology.

[bib26] Fenton MS, Gralla JD (2003). Effect of DNA bases and backbone on sigma70 holoenzyme binding and isomerization using fork junction probes. Nucleic Acids Research.

[bib27] Fenton MS, Lee SJ, Gralla JD (2000). *Escherichia coli* promoter opening and -10 recognition: mutational analysis of sigma70. The EMBO Journal.

[bib28] Gaal T, Ross W, Estrem ST, Nguyen LH, Burgess RR, Gourse RL (2001). Promoter recognition and discrimination by EsigmaS RNA polymerase. Molecular Microbiology.

[bib29] Goldman SR, Ebright RH, Nickels BE (2009). Direct detection of abortive RNA transcripts in vivo. Science.

[bib30] Gries TJ, Kontur WS, Capp MW, Saecker RM, Record MT (2010). One-step DNA melting in the RNA polymerase cleft opens the initiation bubble to form an unstable open complex. Proceedings of the National Academy of Sciences of the USA.

[bib31] Gruber TM, Bryant DA (1997). Molecular systematic studies of eubacteria, using sigma70-type sigma factors of group 1 and group 2. Journal of Bacteriology.

[bib32] Gruber TM, Gross CA (2003). Multiple sigma subunits and the partitioning of bacterial transcription space. Annual Review of Microbiology.

[bib33] Grünberg S, Hahn S (2013). Structural insights into transcription initiation by RNA polymerase II. Trends in Biochemical Sciences.

[bib34] Haugen SP, Berkmen MB, Ross W, Gaal T, Ward C, Gourse RL (2006). rRNA promoter regulation by nonoptimal binding of σ region 1.2: an additional recognition element for RNA polymerase. Cell.

[bib35] Helmann JD (2002). The extracytoplasmic function (ECF) sigma factors. Advances in Microbial Physiology.

[bib36] Helmann JD, Chamberlin MJ (1988). Structure and function of bacterial sigma factors. Annual Review of Biochemistry.

[bib37] Heyduk E, Kuznedelov K, Severinov K, Heyduk T (2006). A consensus adenine at position -11 of the nontemplate strand of bacterial promoter is important for nucleation of promoter melting. The Journal of Biological Chemistry.

[bib38] Johnson JM, Mason K, Moallemi C, Xi H, Somaroo S, Huang ES (2003). Protein family annotation in a multple alignment viewer. Bioinformatics.

[bib39] Juang YL, Helmann JD (1994). A promoter melting region in the primary sigma factor of *Bacillus subtilis*. Identification of functionally important aromatic amino acids. Journal of Molecular Biology.

[bib40] Kapanidis AN, Margeat E, Ho SO, Kortkhonjia E, Weiss S, Ebright RH (2006). Initial transcription by RNA polymerase proceeds through a DNA-scrunching mechanism. Science.

[bib41] Karplus PA, Diederichs K (2012). Linking crystallographic model and data quality. Science.

[bib42] Keilty S, Rosenberg M (1987). Constitutive function of a positively regulated promoter reveals new sequences essential for activity. The Journal of Biological Chemistry.

[bib43] Kenney TJ, York K, Youngman P, Moran CP (1989). Genetic evidence that RNA polymerase associated with sigma A factor uses a sporulation-specific promoter in *Bacillus subtilis*. Proceedings of the National Academy of Sciences of the USA.

[bib44] Kim TK, Ebright RH, Reinberg D (2000). Mechanism of ATP-dependent promoter melting by transcription factor IIH. Science.

[bib45] Ko J, Heyduk T (2014). Kinetics of promoter escape by bacterial RNA polymerase: effects of promoter contacts and transcription bubble collapse. The Biochemical Journal.

[bib46] Kontur WS, Saecker RM, Davis CA, Capp MW, Record MT (2006). Solute probes of conformational changes in open complex (RP o) formation by *Escherichia coli* RNA polymerase at the λ P RPromoter: evidence for unmasking of the active site in the isomerization step and for large-scale coupled folding in the subsequent conversion to RP o†. Biochemistry.

[bib47] Kostrewa D, Zeller ME, Armache K-J, Seizl M, Leike K, Thomm M, Cramer P (2010). RNA polymerase II-TFIIB structure and the mechanism of transcription initiation. Nature.

[bib48] Kouzine F, Wojtowicz D, Arito Y, Resch W, Kieffer-Kwon K-R, Bandle R, Nelson S, Nakahashi H, Awasthi P, Feigenbaum L, Menoni H, Joeijmakers J, Vermeulen W, Ge H, Przytycka TM, Levens D, Casellas R (2013). Global regulation of promotermelting in naeive lymphocytes. Cell.

[bib49] Kulbachinskiy A, Mustaev A (2006). Region 3.2 of the subunit contributes to the binding of the 3′-initiating nucleotide in the RNA polymerase active center and facilitates promoter clearance during initiation. The Journal of Biological Chemistry.

[bib50] Lane WJ, Darst SA (2010). Molecular evolution of multisubunit RNA polymerases: sequence analysis. Journal of Molecular Biology.

[bib51] Lau AY, Schärer OD, Samson L, Verdine GL, Ellenberger T (1998). Crystal structure of a human alkylbase-DNA repair enzyme complexed to DNA: mechanisms for nucleotide flipping and base excision. Cell.

[bib52] Lim HM, Lee HJ, Roy S, Adhya S (2001). A “master” in base unpairing during isomerization of a promoter upon RNA polymerase binding. Proceedings of the National Academy of Sciences of the USA.

[bib53] Liu X, Bushnell DA, Kornberg RD (2011). Lock and key to transcription: sigma-DNA interaction. Cell.

[bib54] Liu X, Bushnell DA, Wang D, Calero G, Kornberg RD (2010). Structure of an RNA polymerase II-TFIIB complex and the transcription initiation mechanism. Science.

[bib55] Lonetto M, Gribskov M, Gross CA (1992). The sigma 70 family: sequence conservation and evolutionary relationships. Journal of Bacteriology.

[bib56] Malhotra A, Severinova E, Darst SA (1996). Crystal structure of a sigma 70 subunit fragment from *E. coli* RNA polymerase. Cell.

[bib57] McClure WR (1980). Rate-limiting steps in RNA chain initiation. Proceedings of the National Academy of Sciences of the USA.

[bib58] McClure WR (1985). Mechanism and control of transcription initiation in prokaryotes. Annual Review of Biochemistry.

[bib59] McClure WR, Cech CL, Johnston DE (1978). A steady state assay for the RNA polymerase initiation reaction. The Journal of Biological Chemistry.

[bib60] McCoy AJ, Grosse-Kunstleve RW, Adams PD, Winn MD, Storoni LC, Read RJ (2007). Phaser crystallographic software. Journal of Applied Crystallography.

[bib61] Mekler V, Pavlova O, Severinov K (2011). Interaction of *Escherichia coli* RNA polymerase σ70 subunit with promoter elements in the context of free σ70, RNA polymerase holoenzyme, and the β′-σ^70^ complex. The Journal of Biological Chemistry.

[bib62] Minchin S, Busby SJW (1993). Location of close contacts between *Escherichia coli* RNA polymerase and guanine residues at promoters either with or without consensus -35 region sequences. The Biochemical Journal.

[bib63] Murakami KS (2013). X-ray crystal structure of *Escherichia coli* RNA polymerase σ70 holoenzyme. The Journal of Biological Chemistry.

[bib64] Murakami KS, Masuda S, Darst SA (2002a). Structural basis of transcription initiation: RNA polymerase holoenzyme at 4 A resolution. Science.

[bib65] Murakami KS, Masuda S, Darst SA (2003). Crystallographic analysis of *Thermus aquaticus* RNA polymerase holoenzyme and a holoenzyme/promoter DNA complex. Methods in Enzymology.

[bib66] Murakami KS, Masuda S, Campbell EA, Muzzin O, Darst SA (2002b). Structural basis of transcription initiation: an RNA polymerase holoenzyme-DNA complex. Science.

[bib67] Nickels BE, Garrity SJ, Mekler V, Minakhin L, Severinov K, Ebright RH, Hochschild A (2005). The interaction between sigma70 and the beta-flap of *Escherichia coli* RNA polymerase inhibits extension of nascent RNA during early elongation. Proceedings of the National Academy of Sciences of the USA.

[bib68] O'Donovan DJ, Stokes-Rees I, Nam Y, Blacklow SC, Schröder GF, Brunger AT, Sliz P (2012). A grid-enabled web service for low-resolution crystal structure refinement. Acta Crystallographica. Section D, Biological Crystallography.

[bib69] Otwinowski Z, Minor W (1997). Processing of X-ray diffraction data collected in oscillation mode. Methods Enzymol.

[bib70] Panaghie G, Aiyar SE, Bobb KL, Hayward RS, de Haseth PL (2000). Aromatic amino acids in region 2.3 of *Escherichia coli* sigma 70 participate collectively in the formation of an RNA polymerase-promoter open complex. Journal of Molecular Biology.

[bib71] Paul BJ, Ross W, Gaal T, Gourse RL (2004). rRNA transcription in *Escherichia coli*. Annual Review of Genetics.

[bib72] Rahman NA (1968). A course in theoretical statistics.

[bib73] Revyakin A, Liu C, Ebright RH, Strick TR (2006). Abortive initiation and productive initiation by RNA polymerase involve DNA scrunching. Science.

[bib74] Roberts J (2006). Biochemistry. RNA polymerase, a scrunching machine. Science.

[bib75] Roe JH, Burgess RR, Record MT (1984). Kinetics and mechanism of the interaction of *Escherichia coli* RNA polymerase with the lambda PR promoter. Journal of Molecular Biology.

[bib76] Ross W, Gourse RL (2009). Analysis of RNA polymerase-promoter complex formation. Methods.

[bib77] Saecker RM, Record MT, deHaseth PL (2011). Mechanism of bacterial transcription initiation: RNA polymerase—promoter binding, isomerization to initiation-competent open complexes, and initiation of RNA Synthesis. Journal of Molecular Biology.

[bib78] Saecker RM, Tsodikov OV, McQuade KL, Schlax PE, Capp MW, Record MT (2002). Kinetic studies and structural models of the association of *E. coli* sigma(70) RNA polymerase with the lambdaP(R) promoter: large scale conformational changes in forming the kinetically significant intermediates. Journal of Molecular Biology.

[bib79] Sainsbury S, Niesser J, Cramer P (2013). Structure and function of the initially transcribing RNA polymerase II-TFIIB complex. Nature.

[bib80] Schickor P, Metzger W, Werel W, Lederer H, Heumann H (1990). Topography of intermediates in transcription initiation of *E.coli*. The EMBO Journal.

[bib81] Schroeder LA, Gries TJ, Saecker RM, Record MT, Harris ME, deHaseth PL (2009). Evidence for a tyrosine–adenine stacking interaction and for a short-lived open intermediate subsequent to initial binding of *Escherichia coli* RNA polymerase to promoter DNA. Journal of Molecular Biology.

[bib82] Schröder GF, Levitt M, Brunger AT (2010). Super-resolution biomolecular crystallography with low-resolution data. Nature.

[bib83] Shultzaberger RK, Chen Z, Lewis KA, Schneider TD (2007). Anatomy of *Escherichia coli* 70 promoters. Nucleic Acids Research.

[bib84] Singh SS, Typas A, Hengge R, Grainger DC (2011). *Escherichia coli* 70 senses sequence and conformation of the promoter spacer region. Nucleic Acids Research.

[bib85] Strong M, Sawaya MR, Wang S, Phillips M, Cascio D, Eisenberg D (2006). Toward the structural genomics of complexes: crystal structure of a PE/PPE protein complex from *Mycobacterium tuberculosis*. Proceedings of the National Academy of Sciences of the USA.

[bib86] Tomsic M (2001). Different roles for basic and aromatic amino acids in conserved region 2 of *Escherichia coli sigma* 70 in the nucleation and maintenance of the single-stranded DNA bubble in open RNA polymerase-promoter complexes. The Journal of Biological Chemistry.

[bib87] Umezawa Y, Nishio M (1998). CH/pi interactions as demonstrated in the crystal structure of guanine-nucleotide binding proteins, Src homology-2 domains and human growth hormone in complex with their specific ligands. Bioorganic & Medicinal Chemistry.

[bib88] Vassylyev DG, Sekine S-I, Laptenko O, Lee J, Vassylyeva MN, Borukhov S, Yokoyama S (2002). Crystal structure of a bacterial RNA polymerase holoenzyme at 2.6 Å resolution. Nature.

[bib89] Vassylyev DG, Vassylyeva MN, Perederina A, Tahirov TH, Artsimovitch I (2007). Structural basis for transcription elongation by bacterial RNA polymerase. Nature.

[bib90] Waldburger C, Gardella T, Wong R, Susskind MM (1990). Changes in conserved region 2 of *Escherichia coli* sigma 70 affecting promoter recognition. Journal of Molecular Biology.

[bib91] Yang CG, Garcia K, He C (2009). Damage detection and base flipping in direct DNA alkylation repair. Chembiochem.

[bib92] Yi C, Chen B, Qi B, Zhang W, Jia G, Zhang L, Li CJ, Dinner AR, Yang CG, He C (2012). Duplex interrogation by a direct DNA repair protein in search of base damage. Nature Structural & Molecular Biology.

[bib93] Yuzenkova Y, Tadigotla VR, Severinov K, Zenkin N (2011). A new basal promoter element recognized by RNA polymerase core enzyme. The EMBO Journal.

[bib94] Zhang Y, Feng Y, Chatterjee S, Tuske S, Ho MX, Arnold E, Ebright RH (2012). Structural basis of transcription initiation. Science.

[bib95] Zuo Y, Steitz TA (2015). Crystal structures of the *E. coli* transcription initiation complexes with a complete bubble. Molecular Cell.

